# Cross-feeding-based rational design of a probiotic combination of *Bacterides xylanisolvens* and *Clostridium butyricum* therapy for metabolic diseases

**DOI:** 10.1080/19490976.2025.2489765

**Published:** 2025-04-06

**Authors:** Shanshan Qiao, Tao Wang, Jingzu Sun, Junjie Han, Huanqin Dai, Mengxuan Du, Lan Yang, Chun-Jun Guo, Chang Liu, Shuang-Jiang Liu, Hongwei Liu

**Affiliations:** a State Key Laboratory of Mycology, Institute of Microbiology, Chinese Academy of Sciences, Beijing, P. R. China; b Savaid Medical School, University of Chinese Academy of Sciences, Beijing, P. R. China; c State Key Laboratory of Microbial Technology, Shandong University, Qingdao, P. R. China; d Jill Roberts Institute for Research in Inflammatory Bowel Disease, Weill Cornell Medicine, Cornell University, New York, NY, USA; e State Key Laboratory of Microbial Resources, Institute of Microbiology, Chinese Academy of Sciences, Beijing, P. R. China

**Keywords:** *Bacteroides xylanisolvens*, *Clostridium butyricum*, cross-feeding, live biotherapeutic products, LBP, metabolic disease

## Abstract

The human gut microbiota has gained interest as an environmental factor that contributes to health or disease. The development of next-generation live biotherapeutic products (LBPs) is a promising strategy to modulate the gut microbiota and improve human health. In this study, we identified a novel cross-feeding interaction between *Bacteroides xylanisolvens* and *Clostridium butyricum* and developed their combination into a novel LBP for treating metabolic syndrome. Using *in-silico* analysis and *in vitro* experiments, we demonstrated that *B. xylanisolvens* supported the growth and butyrate production of *C. butyricum* by supplying folate, while *C. butyricum* reciprocated by providing pABA for folate biosynthesis. Animal gavage experiments showed that the two-strain combination LBP exhibited superior therapeutic efficacy against metabolic disorders in high-fat diet-induced obese (DIO) mice compared to either single-strain treatment. Further omics-based analyses revealed that the single-strain treatments exhibited distinct taxonomic preferences in modulating the gut microbiota, whereas the combination LBP achieved more balanced modulation to preserve taxonomic diversity to a greater extent, thereby enhancing the stability and resilience of the gut microbiome. Moreover, the two-strain combinations more effectively restored gut microbial functions by reducing disease-associated pathways and opportunistic pathogen abundance. This work demonstrates the development of new LBP therapy for metabolic diseases from cross-feeding microbial pairs which exerted better self-stability and robust efficacy in complex intestinal environments compared to conventional single-strain LBPs.

## Introduction

Metabolic disorders, characterized by metabolic failure, when physiological metabolic pathways are disrupted by an inadequate diet, sedentary lifestyle, and lack of physical activity, and clinical manifested as obesity, diabetes, and dyslipidemia, have become a global health challenge with a significant impact on public health.^1–3^ The human gut harbors trillions of microorganisms, collectively known as the gut microbiota, which play a crucial role in host metabolism, immune function, and overall health.^4^ Recent research has increasingly highlighted the intricate interplay between metabolic diseases and the gut microbiota.^5,6^ Alterations in the composition and function of gut microbiota have been revealed to be associated with either the pathogenesis or the resolution of several metabolic disorders.^7,8^ According to present knowledge, the gut microbiota influence host metabolism through various mechanisms, including the production of functional metabolites such as short-chain fatty acids (SCFAs), folic acid, amino acid derivates and secondary bile acids from either dietary or host-derived compounds, the regulation of energy homeostasis, as well as the direct or indirect modulation of gut barrier integrity, immune response and even host behaviors.^9–11^ These findings have sparked interest in exploring innovative strategies to harness the potential of gut microbes for managing metabolic diseases such as metabolic syndrome, diabetes, nonalcoholic fatty liver disease and cardiovascular disease.^5,12,13^

One promising avenue in the regulation of gut microbiota is the development of live biotherapeutic products (LBPs), which are formulations of living microorganisms, designed to confer health benefits when administered in adequate amounts.^14,15^ So far, LBPs that have been experimentally validated as effective for managing metabolic diseases consist of a range of bacterial strains from not only commonly-used probiotics like *Lactobacillus* and *Bifidobacterium*, but also newly-spotted beneficial gut commensal members such as *Parabacteroides, Akkermansia, Blautia* and *Bacteroides*.^15,16^ Especially, the latter group holds greater promise for the development of clinically applicable LBPs, as its members demonstrated more symptom-specific therapeutic effects and better-defined mechanisms. For example, the gut commensal *Parabacteroides distasonis* was verified by animal trial to alleviate metabolic syndrome via production of succinate and secondary bile acids^17^; *Akkermansia muciniphila* has proven efficacy to improve obesity, diabetes mellitus and hepatic steatosis mediated by production of SCFA, protein 9 (P9) and its outer membrane compounds (Amuc-1100)^18^; *Bacteroides xylanisolvens* exerted anti-nonalcoholic fatty liver disease (NAFLD) effects by increase of folate *in-vivo* and modulating folate-dependent one-carbon metabolisms^19^; and *Blautia wexlerae* ameliorates obesity and type 2 diabetes via producing anti-inflammatory metabolites and remodeling gut microbiota.^20^ Although an increasing number of gut commensal bacteria have demonstrated therapeutic effects by animal gavage experiments, our understanding of the stability and robustness of these effects within complicated intestinal environments of diverse hosts in the real world remains limited.^21^ How these LPBs interact within the complex gut microbiota during administration and how these interactions may influence therapeutic outcomes necessitate further research and validation.

Within the gut microbiota, there exist extensive microbe-microbe interactions such as competition and cooperation, which are pivotal for shaping and sustaining the community structure and function.^22,23^ One intriguing microbial interaction pattern is cross-feeding, a process by which different microbial species exchange metabolites, such as nutrients, cofactors and signaling molecules, to support and/or regulate each other’s growth and function.^24^ Such inter-species cooperation in the gut microbiota has significant physiological implications, benefiting hosts by maintaining a stable microbial community, regulating nutrient absorption, producing beneficial metabolites, facilitating probiotic colonization, and resisting pathogen invasion.^22,23^ Recent studies have shed light on how specific pairings of microbial species can synergistically enhance their survival and functionality.^24^ During the process, various metabolites such as short-chain fatty acids (SCFAs), vitamins, amino acids, hydrogen and secondary bile acids are identified to be commonly exchanged among gut microbes.^24,25^ For example, the predominant human gut butyrate-producing bacteria represented by Lachnospiraceae and Clostridiaceae are biotin, thiamine, or folate auxotrophs, and depend on dietary vitamins or cross-feeding from other gut microbes like *Bacteroides* for their survival.^26^ In another example, *Bifidobacterium adolescentis*, an acetate-producer, promotes the growth of Faecalibacterium prausnitzii by supplying acetate as a carbon source, which then benefits hosts by producing butyrate from acetate.^27^ Similarly, *Faecalibacterium prausnitzii* and *Desulfovibrio pigerengage* also engage in a cross-feeding interaction. *D. pigerengage* utilizes lactic acid produced by *F. prausnitzii* to generate acetic acid, which, in turn, supports the growth of *F. prausnitzii* and facilitates the production of butyrate.^28^ Exploring and understanding more cross-feeding interactions is crucial, as it provides insights into how the gut microbiota can be manipulated to promote health or treat diseases, while also facilitating the development of new LBPs from cross-feeding microbial pairs with better self-stability and robust efficacy in complex intestinal environments compared to traditional single-strain LBPs.^28^

In this study, we uncovered a novel cross-feeding interaction between *Bacteroides xylanisolvens* and *Clostridium butyricum*, and developed the newly-identified cross-feeding pair into a novel LBP for treating metabolic disorders. To be specific, we primarily verified by in-silico analysis and *in vitro* experiments that *B. xylanisolvens* supported the growth and butyrate production of *C. butyricum* by supplying folate, while *C. butyricum* reciprocated by providing pABA for folate biosynthesis. Then, animal gavage experiments revealed that the two-strain combination LBP exhibited superior therapeutic efficacy compared to single-strain LBPs containing either *B. xylanisolvens* or *C. butyricum*. Further metagenomic and metabolomic analyses demonstrated that the two-strain combination LBP restored the gut microbial composition and function without causing significant disruptions, thereby sustaining the stability and robustness of the gut microbiota to a greater extent.

## Results

### *Gavage of folate-producing* Bacteroides xylanisolvens *specifically enriched* Clostridium *species in DIO mice*

**Figure 1.**
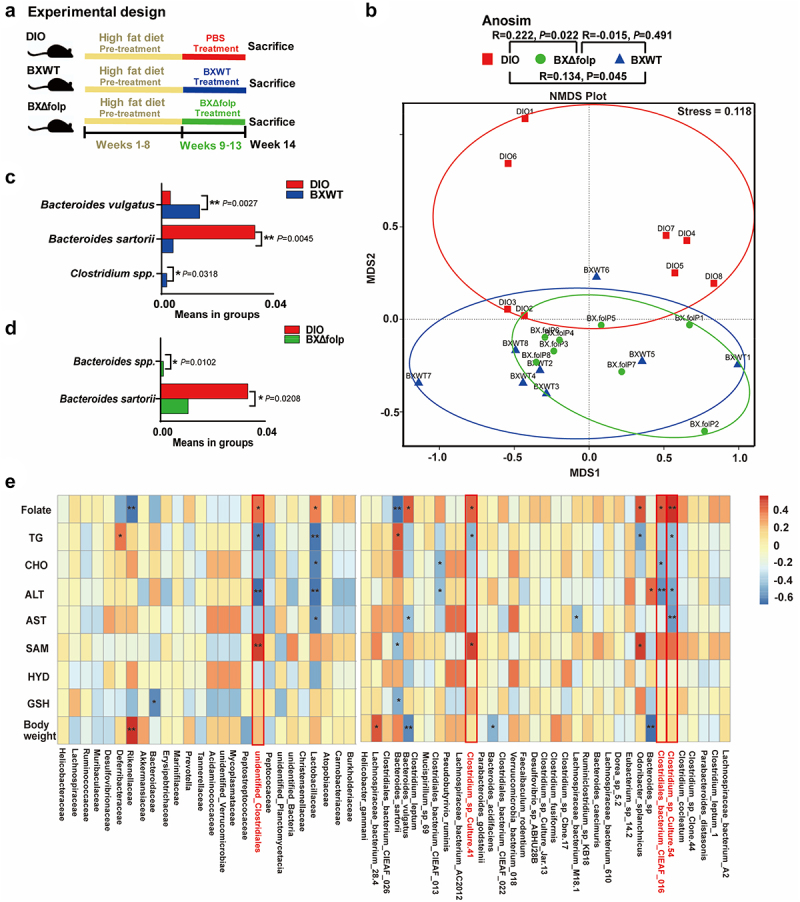
Enrichment of gut *Clostridium spp*. enriched by gavage of folate-producing *Bacteroides xylanisolvens* in DIO mice.

In our previous research, we have demonstrated that the *B. xylanisolvens* alleviated host metabolic disorders via endogenous production of folate.^19^ It is known that microbial biosynthesis of folate requires a complex pathway involving 16 enzymatic processes (Figure S1). However, genomic analysis revealed that *B. xylanisolvens* lacks the *aroD* gene (encoding 3-dehydroquinate dehydratase), rendering it incapable of producing pABA, which is an indispensable precursor for folate biosynthesis. We therefore speculated that *B. xylanisolvens* might obtain pABA from external sources such as cross-feeding nutrients from other gut commensals to enable *in vivo* production of folate. To explore potential collaborators with *B. xylanisolvens* for synergistic folate production, we monitored changes of the gut microbiota in DIO mice administrated with wildtype *B. xylanisolvens* (BXWT) and its folate deficiency mutant BXΔfolp (Figure 1a–d) and analyzed its association with the improved metabolic phenotypes reported in our previous work (Figure 1e). We determined that both the BXWT and BXΔfolp successfully colonized in DIO mice after 4-week gavage (Figure S2A). The 16S rRNA gene amplicons of cecum contents were sequenced and analyzed as described in Methods. According to the Shannon index, the administration of neither BXWT nor the BXΔfolp affected the α diversity of gut microbiota (Figure S2B). However, the gavage of living bacteria significantly changed the taxonomic composition of gut microbiota as the nonmetric multidimensional scaling (NMDS) analysis demonstrated that the β diversity of control group differed from that of BXWT and BXΔfolp treated DIO mice (Figure 1(b)). The linear discriminant analysis effect size (LEfSe) analysis further displayed that *B. vulgatus*, *B. sartorii*, and unclassified *Clostridium* species (*Clostridium spp*.) were increased in BXWT-treated DIO mice (Figure 1(c)), whereas unclassified *Bacteroides* species, and *B. sartorii* were increased in BXΔfolp-treated DIO mice (Figure 1(d)). We then computed the Spearman correlations between the gut microbial taxa that were significantly altered by *B. xylanisolvens* treatment and the improved metabolic phenotypes observed after treatment. It was revealed that four *Clostridium* species were positively correlated with serum folate levels but negatively correlated with TG, T-CHO, ALT, or AST (Figures 1(e) and S3). Noticeably, all the corelated *Clostridium* species were specifically increased by gavage of BXWT rather than the BXΔfolp (Figure 1(c,d)). Above results suggested the existence of an interplay between *B. xylanisolvens*, *Clostridium* species and folate production.

a. Schematic of the experiment. 8-week-old C57BL/6J male mice fed a high-fat diet (60 kcal% Fat, Research diets, D12492) for 8 weeks were sorted into three groups (*n* = 8 each) and orally given 2 × 10^8^ cfu of live *B. Xylanisolvens* or folate synthesis-deficient *B. xylanisolvens* in 0.2 mL of sterile anaerobic PBS daily for 4 weeks.

b. Anosim analysis of differences and nonmetric multidimensional scale (NMDS) distribution of bacterial communities between groups, each dot represents one mouse.

c. LefSe analysis between DIO and BXWT groups.

d. LefSe analysis between DIO and BXΔfolP groups.

e. Heatmap of family, genus, or species-level associations with biochemical indexes, red is positive and blue is negative.

****P < 0.0001, ***P < 0.001,**P < 0.01, *P < 0.05. Abbreviation: DIO, high fat diet-induced obese mice; BXWT, *B. xylanisolvens*; BXΔfolP, *B. xylanisolvens* with knockout of *folP* gene.

### *Identification of cross-feeding between* Bacteroides xylanisolvens *and* Clostridium butyricum

**Figure 2.**
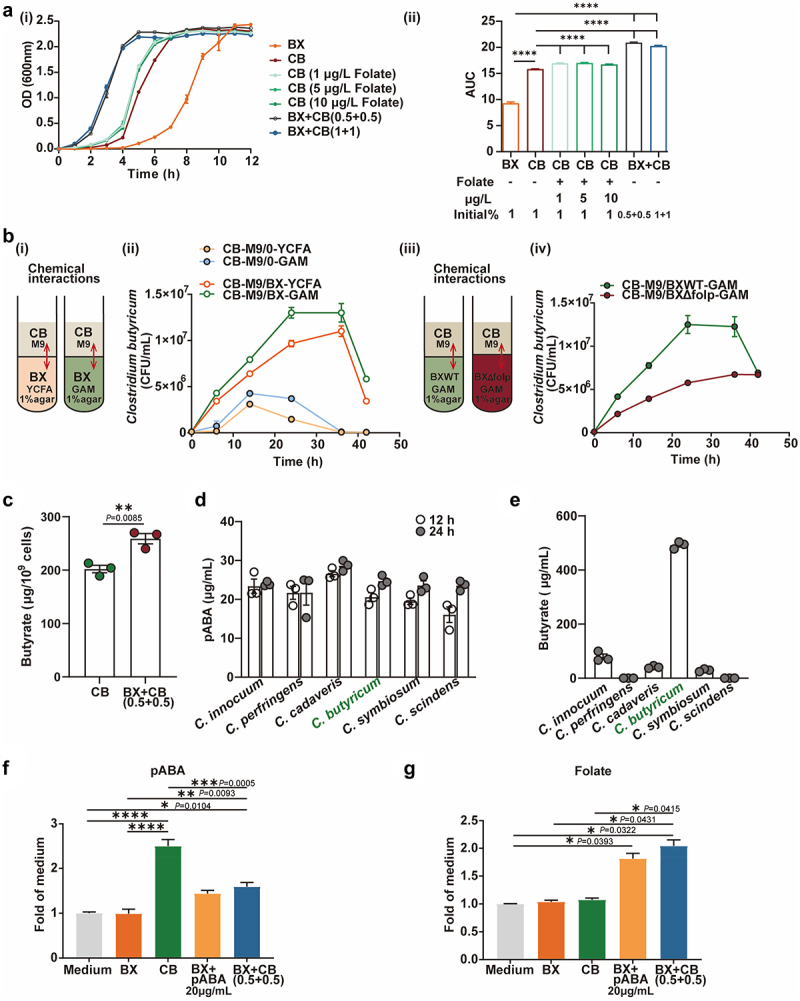
Identification of cross-feeding between *Bacteroides xylanisolvens* and *Clostridium butyricum*.

According to previous studies, some members of gut commensal *Clostridium* are folate auxotroph.^26^ Therefore, our first hypothesis is that the enrichment of gut *Clostridium* species in DIO mice gavaged with wildtype *B. xylanisolvens* was due to the endogenous production of folate. To validate this hypothesis, we designed a series of culture-based *in vitro* experiments using *B. xylanisolvens* and *C. butyricum*- one of the most prevalent and well-characterized gut commensal *Clostridium* species, as a representative of *Clostridium* species. We observed that *C. butyricum* displayed an enhanced growth rate when cultivated in a medium supplemented with folate or when co-cultured with *B. xylanisolvens* (Figure 2(a)). In the case of co-cultivation, the biomass was significantly higher than that of the monoculture of either *B. xylanisolvens* or *C. butyricum*. To further confirm whether *B. xylanisolvens* promoted the growth of *C. butyricum* through cross-feeding folate, we conducted a non-contact co-culture experiment as demonstrated in Figure 2(bi,biii). The results depicted in Figure 2(bii) validated that the biomass of *C. butyricum* in the upper M9 liquid medium increased significantly when *B. xylanisolvens* was present in the culture medium at the bottom. We then confirmed that the cross-feeding nutrient was folate since the Δ*folp* mutant fail to promote the growth of *C. butyricum* (Figure 2(biv)). In addition to promoting the growth of *C. butyricum*, *B. xylanisolvens* also enhanced the production of butyric acids (Figure 2c), as the yield of butyrate per 10^9^ cells in co-culture (259 μg/10^9^ cells) was significantly higher than that of *C. butyricum* monoculture (202 μg/10^9^ cells).

a. The growth curve (i) and area under the curve (AUC) (ii) under different groups (BX, *B. xylanisolvens* was growth in YCFA medium, the initial cell density 1 × 10^6^cfu/mL; CB, *C. butyricum* was growth in YCFA medium, the initial cell density 1 × 10^6^ cfu/mL; CB (1 μg/L folate), *C. butyricum* was growth in 1 μg/L folate YCFA medium, the initial cell density 1 × 10^6^ cfu/mL; CB (5 μg/L folate), *C. butyricum* was growth in 5 μg/L folate YCFA medium, the initial cell density 1 × 10^6^ cfu/mL; CB (10 μg/L folate), *C. butyricum* was growth in 10 μg/L folate YCFA medium, the initial cell density 1 × 10^6^ cfu/mL; BX+CB(0.5 + 0.5), *B. xylanisolvens*and *C. butyricum* was growth in YCFA medium, the initial cell density 5 × 10^5^ cfu/mL and 5 × 10^5^ cfu/mL, respectively; BX+CB(1 + 1), *B. xylanisolvens*and *C. butyricum* was growth in YCFA medium, the initial cell density 1 × 10^6^ cfu/mL and 1 × 10^6^ cfu/mL, respectively).

b. Diagram of bacterial *in vitro* cross-feeding culture, (i) *B. xylanisolvens* was embedded at 5 × 10^6^ cfu/mL or not in 5 mL pre-reduced YCFA or GAM liquid media supplemented with 1% agar at the bottom of an anaerobic tube, and *C. butyricum* was overlaid above it at 1 × 10^6^ cfu/mL in 5 mL pre-reduced M9 minimal medium; (ii) Levels of live *C. butyricum* after incubation in M9 minimal media overlaid on YCFA or GAM agar containing *B. xylanisolvens* or no bacteria (0), *n* = 3; (iii) *B. xylanisolvens* wild-type or knockout of *folP*gene was embedded at 5 × 10^6^ cfu/mL in 5 mL pre-reduced GAM liquid media supplemented with 1% agar at the bottom of an anaerobic tube, and *C. butyricum* was overlaid above it at 1 × 10^6^ cfu/mL in 5 mL pre-reduced M9 minimal medium; (iv) Levels of live *C. butyricum* after incubation in M9 minimal media overlaid on GAM agar containing *B. xylanisolvens* wild-type (BXWT) or *folp*mutant (BXΔfolP), *n* = 3.

c. The levels of butyrate in CB or BX+CB(0.5 + 0.5), samples were collected at 12 h, CB, *C. butyricum* was growth in YCFA medium, the initial cell density 1 × 10^6^ cfu/mL; BX+CB(0.5 + 0.5), *B. xylanisolvens*and *C. butyricum* was growth in YCFA medium, the initial cell density 5 × 10^5^ cfu/mL and 5 × 10^5^ cfu/mL, respectively, *n* = 3.

d. Production of pABA by several *Clostridium spp*.(*C. innocuum*; *C. perfringens*; *C. cadaveris*; *C. butyricum*; *C. symbiosum*; *C. scindens*), bacteria were cultured in YCFA medium, the initial cell density 1 × 10^6^ cfu/mL, and samples were collected at 12 and 24 h, *n* = 3.

e. Production of butyrate by several *Clostridium spp*.(*C. innocuum*; *C. perfringens*; *C. cadaveris*; *C. butyricum*; *C. symbiosum*; *C. scindens*) bacteria were cultured in YCFA medium, the initial cell density 1 × 10^6^ cfu/mL, and samples were collected at 12 h, *n* = 3.

The levels of pABA (f) or folate (g) under different groups, samples were collected at 12 h, Medium, GAM liquid culture control; BX, *B. xylanisolvens* was growth in GAM medium, the initial cell density 1 × 10^6^cfu/mL; CB, *C. butyricum* was growth in GAM medium, the initial cell density 1 × 10^6^ cfu/mL; BX+pABA 20 μg/mL, *B. xylanisolvens* was growth in 20 μg/mL pABA GAM medium, the initial cell density 1 × 10^6^ cfu/mL; BX+CB(0.5 + 0.5), *B. xylanisolvens*and *C. butyricum* was growth in GAM medium, the initial cell density 5 × 10^5^ cfu/mL and 5 × 10^5^ cfu/mL, respectively, *n* = 3.

Data in (a)-(g) are shown as mean ± SEM. Statistical analysis was performed using one-way ANOVA with Tukey’s test in Aii, F, G, unpaired two-tailed Student’s t-test in c, ****P <0.0001, ***P <0.001,**P <0.01, *P <0.05.

Moreover, we observed a positive correlation between *in vivo* folate concentration and *Clostridium* species in the animal trial (Figure 1(e)). Given that *B. xylanisolvens* had a pABA deficiency, we deduced that the *Clostridium* species facilitated *B. xylanisolvens* in folate production via supplying pABA. We therefore analyzed the genomes of six different *Clostridium* species, they all harbored the complete pathway for the biosynthesis of pABA (Table S1). Later *in vitro* fermentation experiment revealed that six tested *Clostridium* species (*C. innocuum*, *C. perfringens*, *C. cadaveris*, *C. butyricum*, *C. symbiosum*, *C. scindens*) produced pABA with a concentration ranging from 16.05 to 28.70 μg/mL (Figure 2(d)). We then took the high butyrate producer *C. butyricum* (Figure 2(e)) as a representative *Clostridium* species to co-culture with *B. xylanisolvens*, and monitor the production of pABA and folate. As shown in Figure 2(f), pABA was significantly accumulated in the monoculture of *C. butyricum* but was consumed when the *C. butyricum* was co-cultured with *B. xylanisolvens*. As a result, the yield of folate was significantly increased in the co-culture compared to the monoculture of *B. xylanisolvens* (Figure 2(g)). Above evidence revealed a new cross-feeding pair of gut commensal microbes. Specifically, *B. xylanisolvens* promoted the growth of *C. butyricum* through cross-feeding of folate, whereas the latter species facilitated folate production of the former via cross-feeding of pABA. To test if the newly-identified cross-feeding mechanism is ubiquitous among *Clostridium* genus, we randomly selected four strains from of *C. innocuum*, *C. symbiosum*, and *C. scindens*, in addition to *C. butyricum* to conducted the co-culture experiment with *B. xylanisolvens*. It revealed that, besides *C. butyricum*, *C. scindens* also exhibited growth-promoting effect (Figure S4).

### *Synergistical reinforcement of* in vivo *anti-metabolic syndrome effect of* B. xylanisolvens *and* C. butyricum *combination therapy*

**Figure 3.**
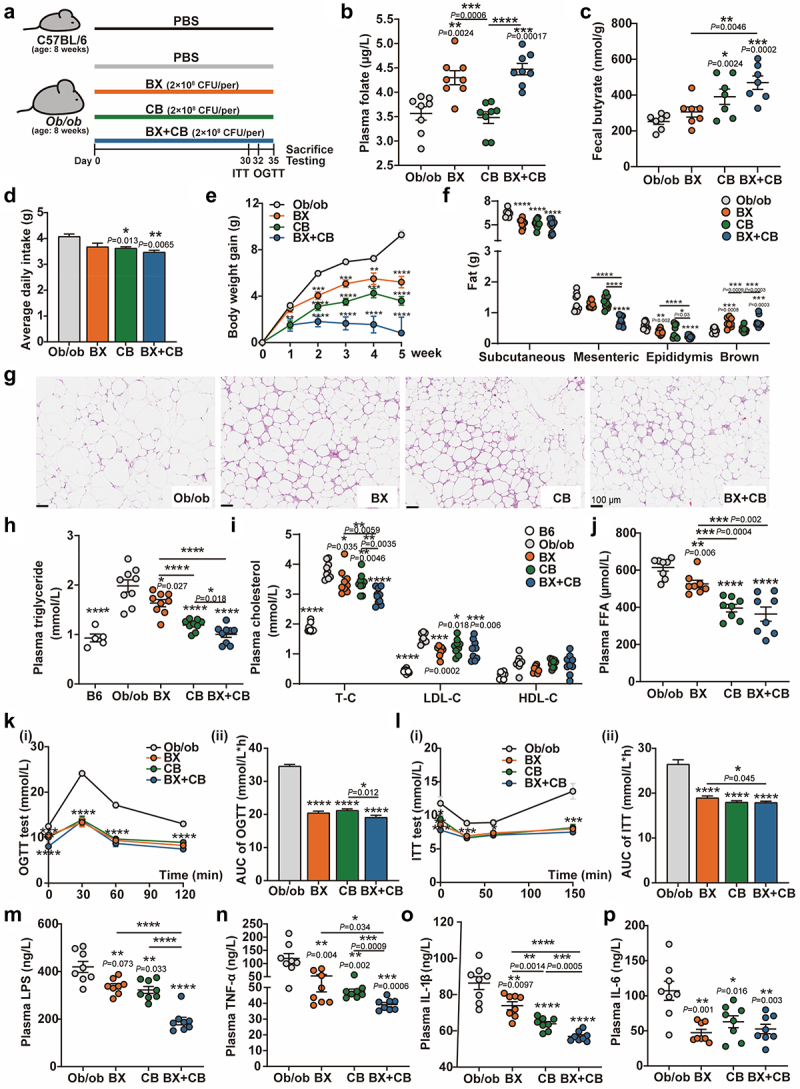
Synergistical reinforcement of *in vivo* anti-metabolic syndrome effect of *B. xylanisolvens* and *C. butyricum* combination therapy.

Previous research conducted by our team and others has reported *that B. xylanisolvens* and *C. butyricum* exhibit probiotic effects to host metabolism through the production of folate and butyrate, respectively.^19,29^ Based on the newly-characterized phenotype in which the cross-feeding pair of *B. xylanisolvens* and *C. butyricum* exhibited higher yields of both folate and butyrate, we developed a two-species probiotic combination therapy for the treatment of metabolic disorders and tested its efficacy using an *ob/ob* mouse model. As displayed in Figure 3(a), the *ob/ob* mice were grouped into four groups and daily gavaged with either PBS (*ob/ob*, *n* = 10), *B. xylanisolvens* (BX, *n* = 10), *C. butyricum* (CB, *n* = 10) or two-strain combination (BX+CB, *n* = 10) for five weeks. The *C57BL/6J* mice treated with PBS were used as a control group (C57, *n* = 10). We initially measured the concentrations of folate and butyric acid in the *ob/ob* mice from each group. It was observed that both *B. xylanisolvens* and the two-strain combination significantly increased plasma folate levels, with the latter showing a more pronounced effect (Figure 3(b)). On the other hand, *C. butyricum* and the two-strain combination significantly increased the fecal butyrate levels to a similar extent (Figure 3(c)). We subsequently assessed the therapeutic effects against metabolic syndromes. In summary, the administration of either *B. xylanisolvens*, *C. butyricum*, or the two-strain combination significantly ameliorated obesity and related metabolic disorders such as hyperlipidemia, hyperglycemia, lipid droplet size, and inflammatory factors in the *ob/ob* mice, albeit to varying degrees (Figure 3(d–q)). To be specific, after a 5-week gavage of *B. xylanisolvens*, *C. butyricum*, and the two-strain combination, the body weight gain of *ob/ob* mice decreased by 44%, 61%, and 91%, respectively (Figure 3(e), Figure S5A). LEE’s index decreased by 44%, 61%, and 91%, accordingly (Figure S5B). Subcutaneous adipose tissue decreased by 20%, 21%, and 24%; mesenteric adipose tissue decreased by 5%, 1%, and 44%; and epididymal adipose tissue decreased by 33%, 33%, and 61%, respectively (Figure 3(f)). Notably, the brown adipose tissue was increased in *ob/ob* mice treated with both *B. xylanisolvens* and two-strain combination by 55% and 60%, respectively (Figure 3(f)). The later H&E staining of white adipose tissues corroborated the fat-reduction effect of microbial treatments as the lipid droplet size in each intervention group was smaller than that of *ob/ob* mice treated with PBS (Figure 3(g), Figure S5F). Regarding lipid metabolism, the level of plasma total triglycerides was reduced by 17%, 40%, and 49% in the *ob/ob* mice treated with *B. xylanisolvens*, *C. butyricum*, and their combination, respectively (Figure 3(h)). Similarly, level of plasma total cholesterol was reduced by 10%, 10%, and 22%, and that of low-density lipoprotein cholesterol decreased by 29%, 19%, and 26%, respectively (Figure 3(i)). The level of plasma free fatty acids (FFA) reduced by 14%, 36% and 41%, respectively (Figure 3(j)). As for hepatic total triglycerides, cholesterol, and liver index, they also exerted a significant decrease trend following administration of three groups of LBPs (Figs. S5C-S5E). As indicated by the results of oral glucose tolerance tests (OGTT) (Figure 3(ki)) and insulin tolerance test (ITT) (Figure 3(li)), LBP treatment significantly alleviated the pathoglycemia of *ob/ob* mice, as the AUC of OGTT (Figure 3(kii)) decreased by 40%, 39%, 45% and the AUC of ITT (Figure 3(lii)) decreased by 28%, 32%, 32%, respectively (Figure 3(k,l)). The blood glucose decreased by 17%, 25%, 38%, respectively, after 30 days of LBP administration (Figure S5G). Additionally, inflammation in *ob/ob* mice was significantly alleviated by microbial treatment, as evidenced by the reduction in plasma levels of LPS by 19%, 23%, and 54%, TNF-α by 56%, 60%, and 67%, IL-1β by 14%, 26%, and 34%, and IL-6 by 56%, 41%, and 51%, respectively, in three treatment groups compared to the PBS-treated control group (Figure 3(m–p)). Besides, *ob/ob* mice was also used as a model of delayed gastric emptying.^30^ To assess if the LBPs have efficacy on stool consistency, frequency, and gut motility, we measured the stool wet weight, and stool water content, the total gastrointestinal (GI) transit time, respectively. The results revealed that *B. xylanisolvens*, *C. butyricum*, and combination treatment significantly improved the fecal characters and gastrointestinal motility function in *ob/ob* mice (Figs. S6A-S6C). Correspondingly, as evidenced by the histological examination of the ileum (Figure S6D), the mucosal barrier was greatly improved, characterized by an increased number of goblet cells.

a. Schematic of the experiment. b. Plasma folate.

c. Fecal butyrate.

d. The average daily food intake.

e. Body weight gain.

f. Changes in several (Subcutaneous/Mesenteric/Epididymis/Brown) fat weight.

g. Size of the adipocytes in the subcutaneous adipose tissue (SAT), representative SAT pathological sections by HE staining; scale bar, 100 μm; magnification, × 20.

h. Plasma triglyceride.

i. Plasma cholesterol (T-C, total cholesterol; LDL-C, low-density lipoprotein cholesterol; HDL-C, high density lipoprotein cholesterol).

j. Plasma free fatty acid (FFA).

k. Blood glucose levels at different time points (**i**) and the area under the curve (AUC) (**ii**) in the OGTT test.

l. Blood glucose levels at different time points (**i**) and the area under the curve (AUC) (**ii**) in the ITT test.

m. Plasma LPS.

n. Plasma TNF-α.

o. Plasma IL-1β.

p. Plasma IL-6.

Abbreviation: B6, negative control group, *C57BL/6J*mice as the wild-type genotype background of *ob/ob* mice; *ob/ob*, modell group, PBS-treated *ob/ob* mice; BX, *B. xylanisolvens*-treated *ob/ob* mice; CB, *C.butyricum*-treated *ob/ob* mice; BX+CB, *B. xylanisolvens* and *C.butyricum*-treated *ob/ob* mice.

Data are presented as the mean ± SEM. *n* = 5-12 mice per group. Statistical analysis was performed using one-way ANOVA followed by the Tukey post hoc test. ****P <0.0001, ***P <0.001, **P <0.01, *P <0.05. Statistical significance between the *ob/ob* (control) group and the three treatment groups is indicated by an asterisk (*) positioned above each treatment group. Additionally, significance between any two treatment groups is marked with a short line and an asterisk (*) above the connecting line.

It was worth noting that, compared to gavage with single *B. xylanisolvens* or *C. butyricum*, the two-strain combination exhibited superior abilities in reducing body weight gain (Figure 3(e), Figure S5A, and Figure S5B), mesenteric and epididymal adipose tissue (Figure 3(f)), total triglycerides (Figure 3(h), Figure S5D), total cholesterol (Figure 3(i), Figure S5E), plasma FFA (Figure 3(j)), plasma LPS (Figure 3(m)), plasma TNF-α (Figure 3(n)), plasma IL-1β (Figure 3(o)) and total GI transit time (Figure S6C). Furthermore, the combination therapy demonstrated superior efficacy compared to *C. butyricum* treatment in improving glucose tolerance and outperformed *B. xylanisolvens* treatment in alleviating insulin resistance (Figure 3(k,l)). These findings confirm that the combination therapy of *B. xylanisolvens* and *C. butyricum* has a more potent anti-metabolic syndrome effect than either strain alone.

### *The coadministration of* B. xylanisolvens *and* C. butyricum *restores the dysbiosis of gut microbiome with less drastic disturbance*

**Figure 4.**
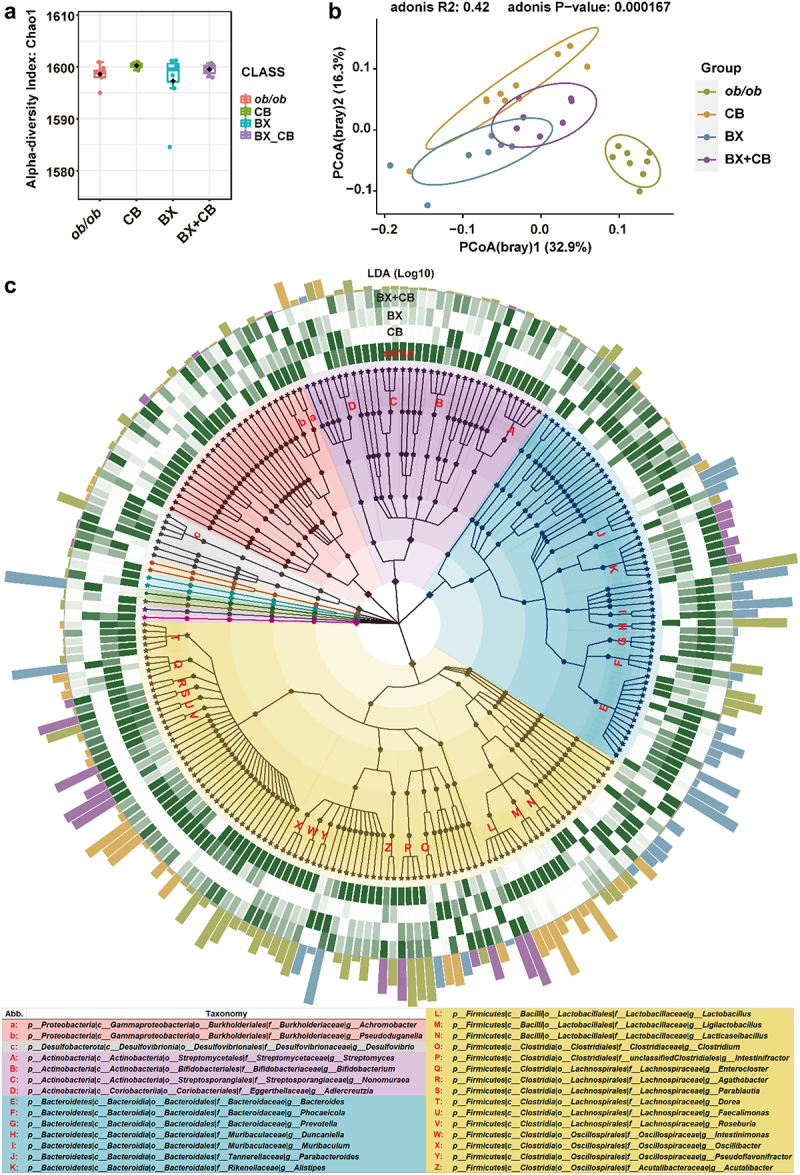
The taxonomic diversity of gut microbiota in *ob/ob* mice treated with vehicle (*ob/ob*), *B. xylanisolvens* (BX), *C. butyricum* (CB), and the mixture of *B. xylanisolvens* and *C. butyricum* (BX+ CB).

**Figure 5.**
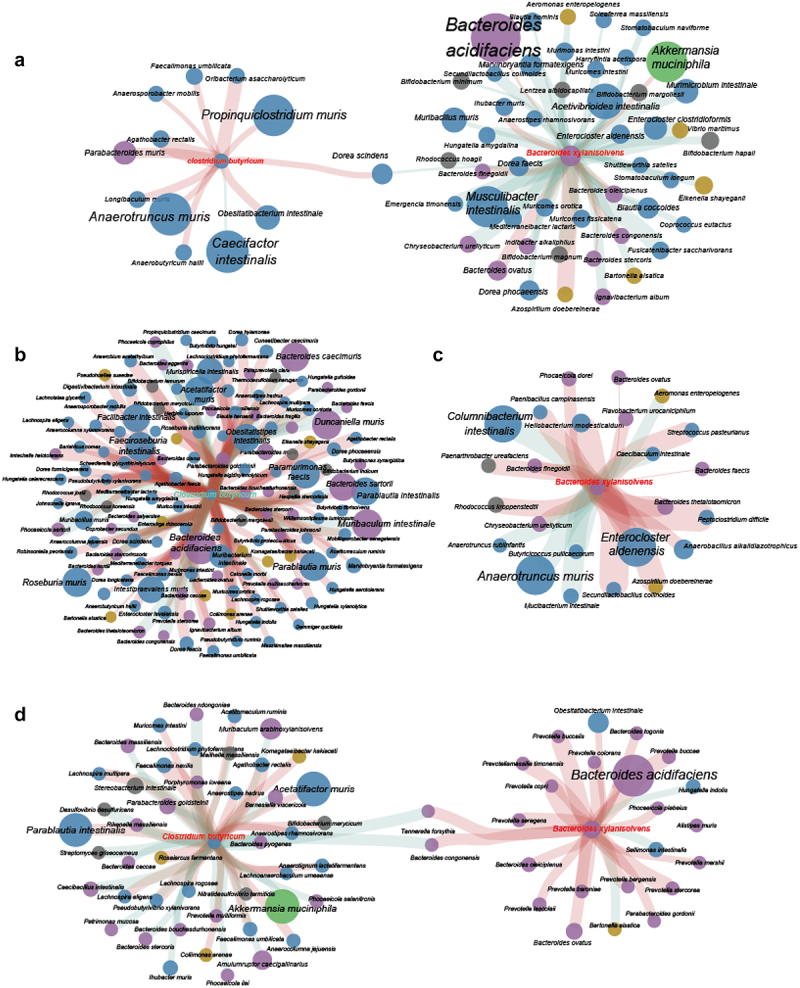
The correlations of *B. xylanisolvens*, *C. butyricum* and their correlated species in in different groups of *ob/ob* mice treated with PBS(A); *C. butyricum* (B); *B. xylanisolvens* (C); the mixture of *B. xylanisolvens* and *C. butyricum* (D); and the metabolite-taxonomy network (E).

We subsequently examined the influence of LBP treatments on the compositions of gut microbiome by profiling the metagenomic of cecal content samples from each experimental group. The taxonomic annotation was performed with Kraken2 as described in Methods. The results revealed that the α diversity of gut microbiota was not influenced by the gavage of either single bacterial strain or the two-strain combination (Figure 4(a)), whereas as the β diversity was modified significantly (Figure 4(b), *p = *0.000167). The Principal Coordinates Analysis (PCoA) demonstrated that the gut microbiota composition in the three LBP-treated groups exhibited greater similarity to each other, while being notably distinct from the model group (Figure 4(b)). The samples from two-strain combination treated group were distributed between those treated with *B. xylanisolvens* and *C. butyricum*, with a slight overlap in their confidence intervals. We then conducted Linear discriminant analysis Effect Size (LEfSe) analysis to identify species that were specifically enriched in each group. As shown in Figure 4(c) and Figure S7, there were a total of 230 species with significantly different abundances among the four groups. Among these, 93 species were specifically enriched in the model group, whereas *C. butyricum* enriched 51 species, *B. xylanisolvens* enriched 49 species, and the two-strain combination group had the lowest number, with only 35 species enriched (Figure S7). Notably, species enriched through gavage with different strains displayed varied taxonomic preferences. Specifically, *C. butyricum* predominantly enriched species from the Firmicutes phylum, while *B. xylanisolvens* treatment primarily enriched species from the Bacteroidetes phylum, followed by Firmicutes phylum. For the two-strain combination treatment, species from Firmicutes and Bacteroidetes were both enriched. At the lower taxon level, *B. xylanisolvens* enriched diverse Bacteroidetes members from Bacteroidaceae, Muribaculaceae, and Tannerellaceae, whereas the two-strain combination treatment specifically enriched species from one *Bacteroidetes* genus- *Alistipes* (Figure 4(c)). In addition, *C. butyricum* and the two-strain combination both enriched taxa from the Lachnospiraceae family, whereas *C. butyricum* exhibited additional preference of more Lactobacillaceae members, a notable lactic acid-producing group within the Firmicutes (Figure 4(c)). We then constructed Spearman correlation networks of the target species (*B. xylanisolvens* and/or *C. butyricum*) with their significantly correlated species (*r*  > 0.7, *p* < 0.05) for different treatment groups. Firstly, we observed that in the gut microbiotas of unmanipulated *ob/ob* mice in model group, *B. xylanisolvens* was associated with 52 species, showing a negative correlation with most species from the Firmicutes phylum and a positive correlation with many species from the Bacteroidetes phylum (Figure 5(a)). In contrast, *C. butyricum* was associated with fewer species (only 12), the vast majority of which belonged to the Firmicutes phylum and were mostly positively correlated (Figure 5(a)). After gavage with *C. butyricum* in mice, the number of species associated with *C. butyricum* significantly increased to 144, most of which belonged to Firmicutes and showed a positive correlation (Figure 5(b)), which was consistent with the specific enrichment of Firmicutes observed in the previous Lefse analysis (Figure 4(c) and Figure S7). On the other hand, only 25 correlated species were identified after *B. xylanisolvens* gavage, with most positively correlated species belonging to the Bacteroidetes phylum and a few to the Firmicutes phylum (Figure 5(c)), which was also in line with the Lefse analysis results (Figure 4(c) and Figure S7). Similarly, after intervention with the two-strain combination, *C. butyricum* still showed more positive correlations with Firmicutes, while *B. xylanisolvens* correlated positively with Bacteroidetes (Figure 5(d)). Namely, species from both Firmicutes and Bacteroidetes were modulated. It appeared that the single strain treatments displayed a more distinct taxonomic preference in modulating the gut microbiota, whereas the combination therapy achieved a more balanced modulation, preserving taxonomic diversity and robustness to a greater extent. Additionally, it is noteworthy that gavage with *C. butyricum* caused the most severe disturbance to the gut microbiota with 144 associated species, and the combination therapy significantly alleviated such disturbance to 49 associated species. Considering that the combination therapy outperformed the single-strain *C. butyricum* intervention by inducing less disruption to the gut microbiota while achieving superior therapeutic effects, it suggested that the profound disturbance of the gut microbiota by *C. butyricum* may not be necessary for its probiotic efficacy but increase the risk of compromising the gut microbiota stability. This result also highlights that the two-strain combination therapy is more effective in targeted restoration of gut microbiota.

a. the Chao 1 index displaying the alpha diversity between groups.

b. the PCoA plot displaying the beta diversity of gut microbiomes in different groups.

c. the cladogram displaying the taxonomic diversity of group-specific taxa identified based on LDA Effect Size (LefSe) analysis. The square node represented phylum, the strike node represented species, and the pentagon node represented genus, family, order and class. The clades and background are color-coded according to different phyla. Background: Yellow: Firmicutes, Blue: Bacteroidetes, Purple: Actinobacteria, Red: Proteobacteria, Grey: Desulfobacterota, Orange: Deinococcota, Cyan: Myxococcota, Olive: Bdellovibrionota, Violet: Chloroflexota, Pink: Spirochaetota. The family-level nodes that included more than 1 genus nodes were marked with letter and the taxonomic information were listed at the bottom of the panel. The bar plot at the outermost layer demonstrated the LDA score (log10) and the color of bars shown in the panel indicated the group of each taxon. The heatmaps at the inner layer displayed the relative abundance of each taxon in four groups (*ob/ob*, BX, CB, BX+CB).

In panel a-d, the color of nodes represented the phylum while the size represented the relative abundance of each species; the thickness of edge represented the Spearman rank correlation coefficient (the cutoff value is 0.7); the red edge represented positive correlations while the green represented negative correlations. In panel e, the red diamond nodes represented significantly changed metabolites, while the pale violet circles represented the species that correlated to each metabolite; the purple edge represented positive correlations between taxon and metabolite while the green ones represented negative correlations. Cytoscape was used for visualization with perfuse force directed layout.

**Figure 6.**
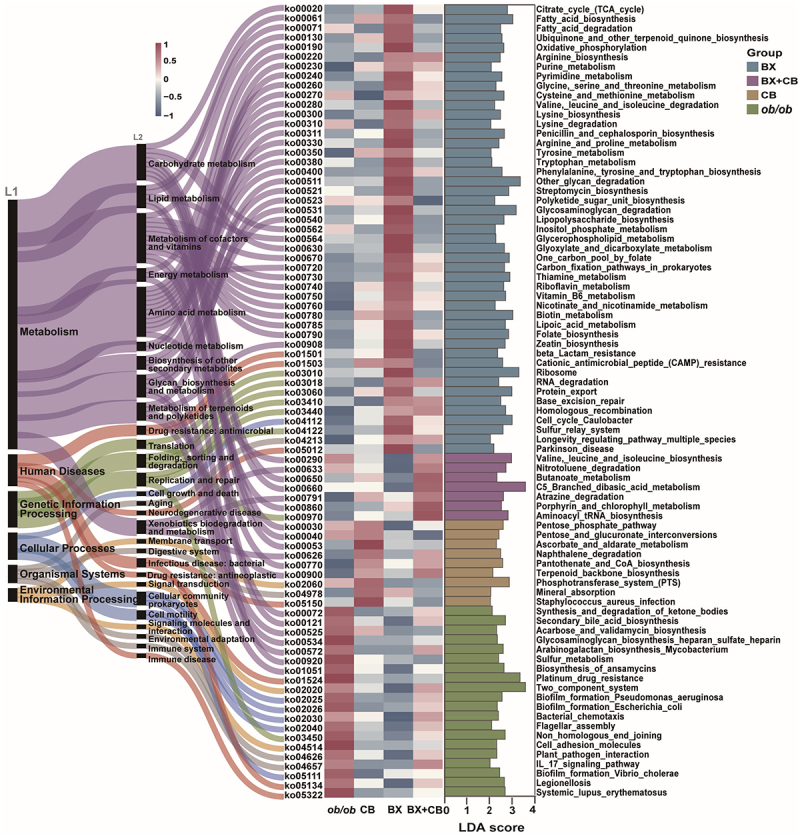
Kegg-based functional differences of gut microbiota among four groups of *ob/ob* mice treated with PBS (*ob/ob*), *B. xylanisolvens* (BX), *C. butyricum* (CB), and the mixture of *B. xylanisolvens* and *C. butyricum* (BX+CB).

To further study the functional implications of the altered gut microbiota driven by gavage, we then annotated the metagenomic raw reads using Humann3 as described in Methods, and the KEGG-based functions displayed as three levels were compared among different groups (Figure 6). At the L1 level, pathways related to cellular processes and human diseases were associated with the PBS-treated *ob/ob* mice, while the most significantly changed pathways in the three LBPs-treated groups were linked to metabolism. At the L2 and L3 levels, gavage with *B. xylanisolvens* increased the abundance of pathways involved in the metabolism of amino acid, cofactor and vitamins. As anticipated, the administration of *B. xylanisolvens* led to a specific enrichment of the folate biosynthesis pathway and the one-carbon pool by folate, and the two-strain combination treatment also notably enhanced folate-related pathways when compared to *C. butyricum* treatment and model group. Different from *B. xylanisolvens*, gavage with *C. butyricum* and two-strain combination mainly increased the levels of carbohydrate metabolism pathways. Interestingly, the enrichment of the butyrate metabolic pathway was most pronounced in the two-strain combination treatment group other than *C. butyricum* treatment. This corresponded to the fact that fecal butyrate concentration was highest in the two-strain combination treatment group (Figure 3(c)). This might be due to the previous observation that two-strain combination enriched mainly butyrate-producing taxa such as Lachnospraceae, whereas *C. butyricum* additionally enriched Lactobacillaceae, which was primarily dominated by lactic acid production (Figure 4(c)). Meanwhile, we measured the colonization of each strain by qPCR analysis of the feces samples collected at the end of animal trial, the relative abundance of *B. xylanisolvens* in the combination group was significantly higher than that in the control group and the two single-strain groups (Figure S8A), also the relative abundance of *C. butyricum* in the combination group and the two single-strain groups was significantly higher than control group (Figure S8B). Moreover, it was worth noting that the model group and single strain treatment groups enriched human disease-related pathways to varying degrees, while the combination group did not exhibit specific enrichment of these pathways. This could be partly explained by the taxonomic composition analysis shown in Figure S8C. The administration of single-strain LBP caused an increase in the relative abundance of the phylum Proteobacteria, which is a primary source of gut opportunistic pathogens (Figure S8C). This increase was statistically significant in *C. butyricum* treated mice and a slight enhancement occurred after *B. xylanisolvens* treatment. On the contrast, the administration of the two-strain combination slightly decreased the relative abundance of Proteobacteria (not significant). These findings corroborated that the combination therapy was more effective in restoring the functional dysbiosis of gut microbiome in *ob/ob* mice.

The Sankey diagram demonstrated the level 1, 2 and 3 class of each enriched pathway in KEGG database; the heatmap displayed the relative abundance (Log10) of each pathway in different groups; the bar chart exerted the LDA score (Log10) analyzed with LEfSe.

### *Metabolic profiles were modulated differently by coadministration of* B. xylanisolvens *and* C. butyricum

**Figure 7.**
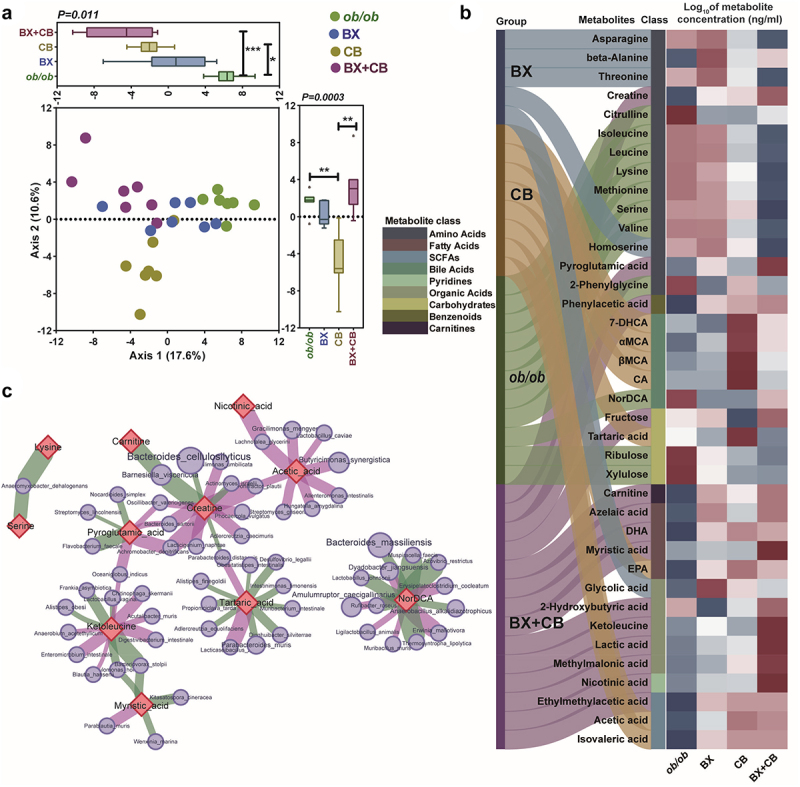
The changes of gut metabolite profiles in high-fat-diet induced mice treated with PBS (ob/ob), B. xylanisolvens (BX), C. butyricum (CB), and the mixture of B. xylanisolvens and C. butyricum (BX+CB).

To further investigate the impact of the three LBP treatment groups on the gut metabolites, the representative cecum samples (*n* = 7 for each group) were tested for quantitative metabolomics. The Partial Least Squares Discriminant Analysis (PLSDA) revealed significant differences in the metabolic profiles among the four groups (Figure 7(a)). Specifically, the gut metabolic composition exhibited the most pronounced dissimilarity in the *C. butyricum* treated mice when compared to the model group (Mod), indicating that *C. butyricum* administration had the greatest impact on the gut metabolic profile, consistent with the observed changes in the gut microbiome as shown in Figures 4(b) and 5(b). Conversely, the *B. xylanisolvens* gavage had the least disruptive effect on the gut metabolic profile, and the combined administration of *B. xylanisolvens* and *C. butyricum* resulted in an intermediate level of disturbance in the composition of gut metabolites, which also aligns with the trends observed in the metagenomic correlation networks shown in Figure 5(c,d). There were in total 163 metabolites of 14 classes detected in the samples (Table S2), with the top metabolite classes were amino acids (22.1%), fatty acids (18.4%), bile acids (17.8%), organic acids (14.1%), carbohydrates (6.1%) and SCFAs (5.5%). We subsequently identified differential metabolites between groups with LefSe, revealing variations in the levels of 38 metabolites spanning 9 distinct classes. Notably, 14 of these metabolites fell within the category of amino acids, constituting 36.8% of the total differential metabolites. Additionally, there were 5 organic acids, 5 bile acids, 4 fatty acids, 4 carbohydrates and 3 SCFAs among the differentially abundant metabolites (Figure 7(b). As displayed by the Sankey-heatmap diagram, the amino acids were enriched in vehicle and *B. xylanisolvens* treated groups. Notably, the branch chain amino acids such as isoleucine, leucin and valine, the enrichment of which were considered as a risk factor of various metabolic disorders,^31,32^ were especially enriched in model group. The administration of either *C. butyricum* or two-strain combination depleted branch amino acids, which might contribute to their metabolically-beneficial effects. Furthermore, as shown in Figure S9, the *C. butyricum* exhibited a significant enrichment of free primary cholic acid (CA, αMCA, βMCA, 7-DHCA), which may be attributed to the bile salt hydrolase activity of *C. butyricum*. According to previous studies,^3,11,17^ the excessive discharge of free primary bile acids into the colorectal region might explain observed significant disturbance of gut microbiota. It is noteworthy that the elevation of free primary cholic acid in the cecum mediated by *C. butyricum* was significantly mitigated when the two-strain combination was administered. This observation aligned with the reduction in gut microbiome disruption, as illustrated in Figure 5(b–d). Moreover, two-strain combination exerted additional enrichments of organic acids, fatty acids, and SCFAs including potentially beneficial metabolites such as DHA, EPA, azelaic acid, nicotinic acid and lactic acid. We next examined the correlations between these altered metabolites and gut microbial species that significantly changed following LBP treatments (*r* > 0.7, *p* < 0.05) with spearman correlation networks (Figure 7(c)). This analysis revealed that the modified levels of 11 metabolites were correlated with abundance variations of multiple gut microbes. This suggests that changes in certain gut metabolites might result from indirect effects mediated by other gut microbes rather than being directly influenced solely by the three LBP administrations.

a. The PLSDA plot displayed the gut metabolite distribution of each group.

b. The Sankey-heatmap diagram displayed the group-specific enrichments of gut metabolites based on LEfSe analysis and the relative abundance of metabolite-correlated pathways in different groups. The Sankey diagrams indicated the metabolites enriched in different groups. The heatmaps showed the concentration of each metabolite; the class of each metabolite was marked with different color bar in the panel.

c. The metabolite-taxonomy correlation network. The red diamond nodes represented significantly changed metabolites, while the pale violet circles represented the species that correlated to each metabolite; the purple edge represented positive correlations between taxon and metabolite while the green ones represented negative correlations. Cytoscape was used for visualization with perfuse force directed layout.

Data in a and b, Kruskal-Wallis test followed by Dunn’s multiple comparisons test were used for statistical analysis; whereas for panel b, only the results of pairwise comparison between Mod and the three groups were marked in the panel; ***p < 0.001, **p < 0.01, *p < 0.05.

## Discussion

Cross-feeding plays indispensable roles in maintaining gut microbiota homeostasis and host health. Microbial communities engage in cross-feeding interactions by sharing metabolites, such as short-chain fatty acids and vitamins, which are essential for the growth and function of members in gut microbiota. These collaborative exchanges prevent the overgrowth of harmful pathogens, enhance the efficiency of nutrient utilization in the complex community, and contribute to the production of signal molecules or the other bioactive metabolites.^24,33^ In this study, we identified a novel gut microbial cross-feeding pair, represented by *B. xylanisolvens* and *C. butyricum*, which increased the production of folate and butyrate through the exchange of pABA and folate. Considering that both folate and butyrate have been proven to benefit host metabolism,^7,10,34–36^ we designed a combined LBP for metabolic disorders using the new cross-feeding pair, and validated its therapeutic effect by animal trials. As our expectation, the co-administration of both strains significantly increased the levels of folate and butyrate *in vivo* (Figure 3(b,c)), meanwhile demonstrating more metabolic benefits compared to a single strain. Our study, on one hand, highlighted the feasibility and advantages of designing and discovering complex LBP based on cross-feeding interactions. While on the other hand, a more in-depth investigation into how the LBP combination influences a broader range of metabolic markers in animal models and potentially in clinical settings is warranted in subsequent research endeavors.

Furthermore, multiple previous cohort studies have indicated alterations in the gut microbiota of individuals with metabolic disorders such as obesity and diabetes, which were characterized by decreased microbial diversity, an increase in opportunistic pathogens from Proteobacteria, an elevated Firmicutes/Bacteroidetes ratio along with a reduction in typical butyrate-producing Firmicutes bacteria and an altered functional profile.^10,19,35,37^ Therefore, we believed that an ideal LBP should regulate host metabolic disorders without cause additional disruptions to the gut microbiota, and what makes an LBP even more desirable is its ability to restore gut microbiota.^21,21^ In this study, though individual administration of either *B. xylanisolvens* or *C. butyricum* alleviated metabolic abnormalities, it also led to varying degrees of adverse disturbances in the gut microbiota. To be specific, both single-strain administrations increased the relative abundance of Proteobacteria and enriched disease-related metabolic pathways (Figure 6 and S8C). The combination therapy, on the other hand, not only exhibited none of above adverse effects, but slightly decreased pathogenesis-related bacterial taxa and pathways. Moreover, gavage with *C. butyricum* particularly exerted the most significant disruption on the gut microbiota by specific increase of Firmicutes group (Figure 5(b) and S8D). It’s noteworthy that although several families within Firmicutes, such as Lachnospiraceae and Clostridiaceae, enriched by *C. butyricum*, are known as major producers of butyrate,^38,39^ the fecal butyrate content in *C. butyricum* treated group was lower compared to the combination therapy group. This observation suggests that the disturbances in gut microbial communities were not directly related to the probiotic effects of LBP but rather were side effects of *C. butyricum* intervention. Such severe disturbances should be given due consideration as they may potentially have detrimental effects on the stability and homeostasis of gut microbiota. Given that *C. butyricum* has been utilized as an LBP in clinical applications,^40,41^ this study serves as a reminder that its potential impact on human gut microbiota composition and bile acid pool should be thoroughly considered, and a careful safety assessment should be conducted. Compared to the single-strain intervention, two-strain combination therapy based on cross-feeding emerged as a more promising and safer LBP for metabolic diseases. This is attributed to the mutual satisfaction of metabolic substrate requirements between *B. xylanisolvens* and *C. butyricum*, resulting in less pronounced disruption of the gut microbiota. Although our study demonstrated the effects of two-strain combination, the precise molecular and physiological pathways mediating these effects warrant further investigation.

Numerous population-based studies have found that serum folate levels are significantly reduced in patients with metabolic diseases such as NAFLD, obesity, and diabetes.^34,36,42^ However, when revisiting the gut metagenomic datasets from different cohort studies,^43–45^ we found that the relative abundance of folate-producing bacterial species like *B. xylanisolvens* and downstream key genes involved in folate synthesis, such as *folp* and *folc*, did not show significant depletion in diseased populations compared to healthy individuals. Furthermore, we have demonstrated in our previous work that *B. xylanisolvens* alleviated NAFLD and related metabolic dysfunctions by enhancing *in vivo* folate level, relying on pABA precursors from other gut bacteria, particularly Clostridium species.^19^ We then investigated that if the pathways involved in pABA biosynthesis are depleted in metabolic disease. In a diabetes cohort study,^43^ we observed a significant depletion of several genes (*aroA, aroB* and *arok)* in the shikimate pathway, which is essential for pABA synthesis, among type 2 diabetes patients (see Supplementary Information Table S6 of reference^43^ for details). This finding suggests that the reduced folate levels in populations with metabolic diseases may be attributed to a decrease of precursors (such as pABA)-producing bacteria participating in the folate synthesis in the gut microbiota. This observation together with this study also underscores the importance of comprehending and implementing cross-feeding interventions for gut microbiota modulation in health management.

We also noted that in addition to the increase of folate and butyrate, the administration of LBPs significantly changed other gut metabolites (Figure 7). It is crucial to recognize that while folate and butyrate are functionally important for metabolic health, the impact of other metabolites induced by the combined LBPs should not be overlooked. This includes bile acids, organic acids such as lactic acid, nicotinic acid, and unsaturated fatty acids such as docosahexaenoic acid (DHA) and eicosapentaenoic acid (EPA), which have been extensively studied and are known to contribute positively to metabolic health.^9,19,46,47^ These indirect metabolite alterations induced by the administration of LBPs might be additional contributors to the observed therapeutic efficacy.

While our study demonstrated the therapeutic potential of the *B. xylanisolvens* and *C. butyricum* combination, it is important to note that neither strain is currently listed as an approved edible probiotic. This presents potential regulatory and safety challenges for clinical application. Further research is essential to evaluate the scalability and practicality of translating the findings from this preclinical research to clinical applications. A comprehensive safety evaluation, long-term effects, and multiple phases of clinical trials are warranted to validate the conclusions drawn from animal experiments. These trials must meticulously determine optimal dosage, formulation, therapeutic indications, pharmacokinetic profiles, and a comprehensive array of additional assessments that are crucial for the successful clinical integration of a therapeutic agent. Additionally, regulatory approval would require rigorous documentation of manufacturing standards, strain stability, and consistent therapeutic efficacy. Moreover, many recent researches have illuminated the potential of LBP to augment the efficacy of pharmaceuticals in managing metabolic disorders, suggesting a promising avenue for therapeutic intervention.^14,28^ However, we should also note that the intricate dynamics of introducing combined LPBs into the complex milieu of the gut’s metabolic and microbial landscape raise the possibility of unforeseen interactions with concurrent medications. These interactions may yield a beneficial amplification, as demonstrated within the scope of this study. Conversely, there is a potential for a detrimental reduction in efficacy due to antagonistic interactions.^48–50^ Elucidating the precise nature of these potential interactions is a critical area for future research, requiring meticulous experimental inquiry and validation to harness the full therapeutic potential of such combinatorial approaches.

In addition to treatment of metabolic diseases, the ability of this combination strains to facilitate folate biosynthesis suggests their potential use in addressing folate deficiencies in specific populations, such as pregnant women, who require active folate supplementation to prevent neural tube defects and support overall health.^51–53^ Additionally, this cross-feeding pair also has potential applications in veterinary medicine and animal husbandry. Folate supplementation in pigs has been shown to enhance immunity, improve reproduction, and reduce the risk of fetal defects.^54–56^ Thus, incorporating *B. xylanisolvens* and *C. butyricum* into livestock management practices could provide a natural, microbiome-driven solution to bolster animal health and economic productivity. However, these applications require further validation across different hosts and environments, considering potential limiting factors such as strain colonization efficiency, competition with native microbiota, and safety. Future research should explore these strains’ diverse applications while emphasizing optimized management and nutritional strategies to maximize health benefits and economic value in both humans and animals.

## Supplementary Material

Methods and SI Figures Revised1211.docx

## Data Availability

All data are available on request from the authors.
