# Supplementary material for: Cross-feeding-based rational design of a probiotic combination of Bacterides xylanisolvens and Clostridium butyricum therapy for metabolic diseases
Source: Gut Microbes. 2025 Apr 6;17(1):2489765. doi: 10.1080/19490976.2025.2489765 (PMC11980479; doi:10.1080/19490976.2025.2489765)
Supplement: Methods and SI Figures Revised1211.docx [file KGMI_A_2489765_SM7540.docx]

Supplementary Materials for:

**Cross-feeding based rational design of a probiotic combination of *Bacterides xylanisolvens* and *Clostridium butyricum* therapy for metabolic diseases**

Shanshan Qiao^1,2^, Tao Wang^1,2^, Jingzu Sun^1,2^, Junjie Han^1,2^, Huanqin Dai^1,2^, Mengxuan Du^3^, Lan Yang^3^, Chun-Jun Guo^5^, Chang Liu^3^ *, Shuang-Jiang Liu^3,4^, Hongwei Liu^1, 2, 6,^*

^1^ State Key Laboratory of Mycology, Institute of Microbiology, Chinese Academy of Sciences, No. 1 Beichenxi Road, Chaoyang District, Beijing, 100101, P. R. China

^2^ Savaid Medical School, University of Chinese Academy of Sciences, Beijing, 100049, P. R. China

^3^ State Key Laboratory of Microbial Technology, Shandong University, Qingdao 266237, P. R. China

^4^ State Key Laboratory of Microbial Resources, Institute of Microbiology, Chinese Academy of Sciences, No. 1 Beichenxi Road, Chaoyang District, Beijing, 100101, P. R. China

^5^Jill Roberts Institute for Research in Inflammatory Bowel Disease, Weill Cornell Medicine, Cornell University, New York, NY 10021, USA

^6^ Lead Contact Tel: +86 10 64806074; [liuhw@im.ac.cn](mailto:liuhw@im.ac.cn) (H.L.)

* Corresponding authors

**Table of Contents**

[Methods 3](#_Toc174964855)

[1. Ethics, animals, and probiotics 3](#_Toc174964856)

[2. Cross-Feeding *in vitro* Assay 4](#_Toc174964857)

[3. *In vitro* liquid culture of *B. xylanisolvens* and *C. butyricum.* 4](#_Toc174964858)

[4. Measurement of Gastrointestinal Transit Times Using Non-absorbable Red Carmine D 5](#_Toc174964859)

[5. Body weight and food intake 5](#_Toc174964860)

[6. Fecal collections 5](#_Toc174964861)

[7. Tissue sampling 5](#_Toc174964862)

[5. Biochemical analysis 5](#_Toc174964863)

[6. Insulin tolerance test (ITT) and oral glucose tolerance test (OGTT) 5](#_Toc174964864)

[7. Histopathological examination and immunohistochemical staining 6](#_Toc174964865)

[8. DNA isolation and qPCR 6](#_Toc174964866)

[9. Generation of the folate synthesis-deficient strain of *B. xylanisolvens* 6](#_Toc174964867)

[10. 16S rRNA gene sequencing and analysis 6](#_Toc174964868)

[11. Targeted metabolomics analyses 7](#_Toc174964869)

[12. Metagenomic sequencing and analysis 8](#_Toc174964870)

[13. Statistical analysis 8](#_Toc174964871)

[Supplementary figures 10](#_Toc174964872)

[Figure S1. The chemical structure of the folate and schematic of bacterial *de novo* folate synthesis. corresponding to Fig. 1. 10](#_Toc174964873)

[Figure S2. The relative abundance and α-diversity of gut microbiota in *B. xylanisolvens* wild-type or *folp* mutant colonized DIO mice, corresponding to Fig. 1. 11](#_Toc174964874)

[Figure S3. The spearman correlation analysis of fecal folate-*B. xylanisolvens*, and *B. xylanisolvens*-*Clostridium spp.*, corresponding to Fig. 1. 12](#_Toc174964875)

[Figure S4. Exploration of cross-feeding between *Bacteroides xylanisolvens* and *Clostridium spp.*, corresponding to Fig. 2. 13](#_Toc174964876)

[Figure S5. Synergistical reinforcement of *in vivo* anti-metabolic syndrome effect of *B. xylanisolvens* and *C. butyricum* combination therapy, corresponding to Fig. 3. 14](#_Toc174964877)

[Figure S6. Synergistical reinforcement of *in vivo* fecal characters and gastrointestinal motility function of *B. xylanisolvens* and *C. butyricum* combination therapy, corresponding to Fig. 3. 16](#_Toc174964878)

[Figure S7. The enriched species in each group by LEfSe analysis, corresponding to Fig. 4. 18](#_Toc174964879)

[Figure S8. The relative abundance of *B. xylanisolvens*, *C. butyricum*, and Proteobacteria in each group. 19](#_Toc174964880)

[Figure S9. The fecal bile acids profiling of *ob/ob* mice treatment with PBS or *C. butyricum*, corresponding to Fig. 7. 20](#_Toc174964881)

# **Methods**

## **1. Ethics, animals, and probiotics**

All animal procedures in this study were performed in accordance with the recommendations in the Guide for the Care and Use of Laboratory Animals of the Institute of Microbiology, Chinese Academy of Sciences (IMCAS) Ethics Committee. The protocols were approved by the Committee on the Ethics of Animal Experiments of IMCAS (permit APIMCAS2020091). Mice were bred under specific-pathogen-free (SPF) conditions in a laboratory animal facility at IMCAS. All animal experiments were conducted under isoflurane anesthesia, and all efforts were made to minimize suffering. C57BL/*6J* mice, *ob/ob* mice (mice homozygous for the obese spontaneous mutation) were purchased from Vital River Laboratory Animal Technology Co., Ltd. (Beijing, China). The 8-week-old mice were raised and housed under SPF conditions individually. All mice had free access to food and water at a 12 h dark-light cycle. The sample sizes for all animal studies are provided in each figure legend. No animals were excluded from this study. The probiotics strain, *Bacterides xylanisolvens* (CGMCC 1.31487) and C*lostridium butyricum* (CGMCC 1.31469), and other *Clostridium spp.*(*C. innocuum*, *C. perfringens*, *C. cadaveris*, *C. symbiosum*, and *C. scidens*) were provided by hGMB^1^. The strains were cultured in YCFA (10 g/L, casitone, 2.5 g/L yeast extract, 5.0 g/L glucose, 45 mg/L MgSO_4_*7 H_2_O, 90 mg/L CaCl_2_*2H_2_O, 0.45 g/L K_2_HPO_4_, 0.45 g/L KH_2_PO_4_, 0.9 g/L NaCl, 1 mg/L resazurin) or GAM (10 g/L casitone, 3 g/L soya petone, 15 g/L proteose peptone, 13.5 g/L digested serum, 5 g/L yeast extract, 2 g/L meta extract, 1.2 g/L liver extract, 3 g/L dextrose, 0.3 g/L soluble starch, 0.5 g/L L-cysteine hydrochloride, 0.5 g/L L-arginine, 0.3 g/L L-tryptophan, 2 g/L NaHCO_3_, 2.5 g/L KH_2_PO4, 3 g/L NaCl, 0.15 g/L CH_2_(SH)COONa, 2.46 g/L CH_3_COONa, 0.01 g/L Hemin, 0.001 g/L Resazurin, 5% Sheep Blood) medium at 37 °C in an anaerobic chamber for 24 h. For the *in vivo* efficacy assay*,* cell pellets were obtained by centrifuging at 8,000 × g for 10 min at 4 °C. The cell suspension for oral administration was prepared by suspending the cultured bacterial cells in oxygen-free PBS with a final cell density of 1×10^9^ cfu/mL, 200 μL of the bacterial suspension was given daily. For the colony-forming unit (CFU) measurement, first to measure the growth curve of each strain, then make a series dilutions from a OD_600_ mesured bacterial culture, do CFU plating and counting, produce a standard curve of OD_600_ plotted against cell count (CFU/mL).

In assays with live *B. xylanisolvens* and folate synthesis-deficient *B. xylanisolvens,* 8-week-old C57BL/6J male mice fed a high-fat diet (60 kcal% Fat, Research diets, D12492) for 8 weeks were sorted into three groups (n = 8 each) and orally given 2 × 10^8^ cfu of live *B. xylanisolvens* or folate synthesis-deficient *B. xylanisolvens* in 0.2 mL of sterile anaerobic PBS daily for 4 weeks.

In assays involving *B. xylanisolvens* (BX), C*. butyricum* (CB), and combined BX+CB, 8-week-old *ob/ob* male mice were sorted into three groups (n = 8 each) based on their blood glucose levels and body weight. Mice in the BX group were treated daily with 2 × 10^8^ cfu of *B. xylanisolvens* in 0.2 mL of sterile anaerobic PBS by mouth. Mice in the CB group were given 2 × 10^8^ cfu of C*. butyricum* in 0.2 mL of sterile anaerobic PBS daily. Mice in the BX+CB group were given 1 × 10^8^ cfu *B. xylanisolvens* and 1 × 10^8^ cfu of C*. butyricum* in 0.2 mL of sterile anaerobic PBS daily. The vehicle group (8-week-old *C57BL/6J* male mice, n=8) was given an equivalent volume of sterile anaerobic PBS. Treatments were continued for 5 weeks.

**2. Cross-Feeding *in vitro* Assay**

Cross-feeding was measured as previously described^2^. *B. xylanisolvens* wild-type or *folp* mutant was embedded at 5x10^6^ cfu/mL in 5 mL pre-reduced YCFA or GAM-based liquid media supplemented with 1% agar at the bottom of an anaerobic tube, and C*. butyricum* was overlaid above it at 1x10^6^ cfu/ml in 5 mL pre-reduced M9 minimal media. CFU plate test confirmed no ectopic translocation of embedded *B. xylanisolvens* from the agar compartment into the above M9 liquid compartment. Co-cultures were placed at 37°C, for each time point, aliquots were taken from the top and bottom compartments, plated in a dilution series in rich medium (BHI) agar (10% sheep blood, 8.0 g/L brain heart infusion from solids, 5.0 g/L peptic digest of animal tissue, 16.0 g/L pancreatic digest of casein, 5.0 g/L sodium chloride, 2.0 g/L glucose, 2.5 g/L disodium hydrogen phosphate, 13.5 g/L agar), and colonies were counted.

**3.** ***In vitro* liquid culture of *B. xylanisolvens* and *C. butyricum.***

0.1 mL of *B. xylanisolvens* (1 × 10^8^ cfu/mL) were inoculated anaerobically into freshly prereduced YCFA medium (10 mL) , the initial cell density 1×10^6^ cfu/mL; 0.1 mL of *C. butyricum* (1 × 10^8^ cfu/mL) were inoculated anaerobically into freshly prereduced YCFA medium (10 mL), the initial cell density 1×10^6^ cfu/mL; 0.05 mL *B. xylanisolvens*  (1 × 10^8^ cfu/mL) mixed 0.01 mL *C.butyricum* (1 × 10^8^ cfu/mL) were inoculated anaerobically into freshly prereduced YCFA (10 mL), the initial cell density 5×10^5^ cfu/mL and 5×10^5^ cfu/mL, respectively; 0.1 mL *B. xylanisolvens* (1 × 10^8^ cfu/mL) mixed 0.1 mL *C. butyricum* (1 × 10^8^ cfu/mL) were inoculated anaerobically into freshly prereduced YCFA (10 mL), the initial cell density 1×10^6^ cfu/mL and 1×10^6^ cfu/mL, respectively. 0.1 mL of *C. butyricum* (1 × 10^8^ cfu/mL) were inoculated anaerobically into freshly prereduced YCFA medium containing folate (10 mL), the initial cell density 1×10^6^ cfu/mL, The YCFA containing folate (the final concentrations are 1 μg/L , 5 μg/L, and 10 μg/L, respectively. The growth kinetics of bacteria were analyzed using a Spectra Max 190 microplate reader (Molecular Devices Inc.) according to the optical density (OD600). The bacterial supernatant were collected on 12 h, and detected SCFAs by GC-MS analysis as described in our early report^3^, folate (JinyoTech, F14536-B), or pABA (MyBio, MBS9366794) by enzyme-linked immunosorbent assay (ELISA) Kit following the manufacturer’s instructions.

*In vitro* culture of *Clostridium spp.*, 0.5 mL of *C. innocuum*, *C. perfringens*, *C. cadaveris*, *C. butyricum*, *C. symbiosum*, and *C. scidens* (1 × 10^8^ CFU/mL) were inoculated anaerobically into freshly prereduced YCFA medium (50 mL). The bacterial supernatant were collected on 12 h and 24h, and detected SCFAs by GC-MS analysis as described in our early report^3^, and pABA (MyBio, MBS9366794) by ELISA Kit.

**4.** **Measurement of Gastrointestinal Transit Times Using Non-absorbable Red Carmine D**

Carmine red (Sigma-Aldrich) was prepared as a 6% (w/v) solution in 0.5% methylcellulose (Sigma-Aldrich). Mice were maintained on a strict 12-hr light cycle (lights on between 06:00 and 18:00) and gavaged with 0.15 mL of the carmine solution between 08:00 and 08:30 local time. Animals were not fasted beforehand. Feces were collected every 30 min (up to 8 hr from time of gavage) and streaked across a sterile white napkin to assay for the presence of the red carmine dye. The time from gavage to initial appearance of carmine in the feces was recorded as the total intestinal transit time for that animal.

## **5. Body weight and food intake**

The body weight was measured every 2-3 days. Food intake per mouse per day was determined by the following equation: (total food intake each cage)/(5 mice per cage) / (days of food consumption).

## **6. Fecal collections**

Animals were kept in an empty cage without bedding for 15 min to gather fresh stool samples into tubes. Tubes were stored at -80 ºC until analysis. Feces wet weight were recorded immediately, after freeze-vacuum drying treatment, feces were reweighing to calculate fecal water content.

## **7. Tissue sampling**

After treatment, animals were anesthetized with isoflurane anesthesia (Beijing Chemical Works, Beijing, China), and blood was sampled from the portal and cava veins. After euthanasia by cervical dislocation, mice were exsanguinated. The intestines, cecum content, adipose, and the liver were precisely dissected, weighed, one piece of adipose and ileum tissue from each mouse were fixed in 10% formalin for histology, tissue immersed immediately in liquid nitrogen, and stored at −80 °C for further analysis.

## **5. Biochemical analysis**

The level of blood glucose was measured using a glucose meter (Accu-Chek, Roche, Switzerland), Levels of plasma TC (Nanjing Jiancheng, A111-1), TG (Nanjing Jiancheng, A110-1), HDL-C (Nanjing Jiancheng, A112-1), LDL-C (Leagene, TC1265); TC, TG, HDL-C, LDL-C in liver were measured by commercial kits. Plasma FFA, TNFα, IL-1β, IL-6, LPS, pABA (My bio, MBS9364277) and total folate (JinyoTech, F14536-B) in the plasma and culture media were quantified using an ELISA kit following the manufacturer’s instructions.

## **6. Insulin tolerance test (ITT) and oral glucose tolerance test (OGTT)**

An ITT was performed by injecting insulin (0.6 U/kg) intraperitoneally after 8 h of fasting. An OGTT was performed by giving a glucose bolus (2 g/kg) by gavage after overnight fasting. The level of blood glucose was measured using a glucose meter (Accu-Chek, Roche, Switzerland) before oral glucose load (0 min) and at 30, 60, and 120 min after injecting insulin or oral glucose load. The AUCs generated from the data collected during the ITT or OGTT were calculated with GraphPad 9.0.

## **7. Histopathological examination and immunohistochemical staining**

Samples of ileum, abdominal white adipose tissue (WAT) were resected and fixed with 10% formaldehyde phosphate-buffered saline (pH 7.4), embedded in paraffin, sectioned, stained with hematoxylin/eosin (H&E), or alcian blue-periodic acid-schiff (AB-PAS) staining and finally analyzed by a Zeiss Imager A2-M2 microscope (Carl Zeiss AG, Cöttingen). Representative slices of Oil Red O-stained liver sections were further scanned with a Zeiss Axio Scan Z1 Scan-Scope scanner.

## **8. DNA isolation and qPCR**

Total bacterial DNA was extracted from mouse feces that were colonized with either the *B.xylanisolvens* wild-type strain or the *folp* mutant using the QIAamp DNA stool mini kit (Qiagen, Inc. 51604). qPCR was performed on a 7500 Fast Real-Time PCR System with various primers (For all bacteria, 16S-F: WACGCGARGAACCTTACC, 16S-R: TGACGGGCGGTGWGTAC; for *B. xylanisolvens-*specific primers, BX-F: ACGCTCGGATCCTCCGTATT, BX-R: AGGATGACTGCCCTATGGGT; for *C. butyricum*-specific primers, CB-F: GTGCCGCCGCTAACGCATTAAGTAT, CB-R: ACCATGCACCACCTGTCTTCCTGCC). The qPCR reaction system contained ~100 ng of DNA, 0.5 μM primers per 0.15 μM probe, and SYBR green chemistry (Kapa Biosystems). PCR amplification was performed using the following cycling parameters: 3 min at 95 °C, 37 cycles of 3 s at 95 °C and 30 s at 60 °C. The copy number was determined from the standard curve generated using a synthetic template.

**9. Generation of the folate synthesis-deficient strain of *B. xylanisolvens***

An internal fragment (514 bp) of the folp gene was cloned into the pGERM suicide vector incorporating *E. coli* (bla) and *B. xylanisolvens* (ermG) selective markers. The resulting construct was transformed into the conjugative *E. coli* WM3064 strain. The *E. coli* donor strain WM3064 and the *B. xylanisolvens* recipient strain were grown and treated with a previously reported method ^4^ to generate the folate synthesis-deficient strain of *B. xylanisolvens*.

## **10. 16S rRNA gene sequencing and analysis**

DNA for gut microbiota analysis was extracted from approximately 50 mg of cecum contents of ob/ob mice treated with probiotic by a previously reported method (Wang et al., 2018). The V3-V4 region of 16S rRNA was amplified using the primers F341 (CCTACGGGRSGCAGCAG) and R806 (GGACTACVVGGGTATCTAATC) by PCR and sequenced in the HiSeq PE250 platform (Illumina) using the 2 x 250 bp paired-end protocol. The obtained pair-end reads were trimmed and then assembled according to PANDAseq^5^. After filtering the chimeras by USEARCH, sequences were clustered into Operational Taxonomic Units (OTUs) at a similarity cutoff value of ≥97% using the UPARSE algorithm. A representative sequence of each OTU was assigned to the taxa at genus level in the optimized version of the RDP database (http://rdp.cme.msu.edu). Each unique OTU was subjected to BLAST against NCBI 16S database to identify closest match to the taxa at species level based on lowest e-value and identity %. Abundances were recovered by mapping the de-multiplexed reads to the UPARSE OTUs. A rarefied OTU table from the output files was further analyzed by a visualization toolkit. The resulting abundance table and taxonomic classification was loaded into R. Statistical analysis of differentially abundant sequences and taxa were performed by DESeq2 1.16.1^6^ and the log_2_ fold changes (log_2_FC) represented the comparison against the reference level.

## **11. Targeted metabolomics analyses**

Feces were thawed on an ice bath to reduce degradation. Approximately 5 mg of each lyophilized sample was weighed and transferred to a new 1.5-mL tube. Next, 25 μL of water was added, and the samples were homogenized with zirconium oxide beads for 3 min. Methanol (120 μL) containing internal standard was added to extract the metabolites. The samples were homogenized for another 3 min, then centrifuged at 1,800 g for 20 min, and 20 μL of supernatant was then transferred to a 96-well plate. The subsequent procedures were performed on an Eppendorf epMotion Workstation (Eppendorf Inc., Humburg, Germany). Freshly prepared derivative reagent (20 μL) was added to each well, then the plates were sealed and derivatized at 30 °C for 60 min. The samples were then further diluted by adding 330 μL of ice-cold 50% methanol solution, and the plates were stored at -20 °C for 20 min, then centrifuged at 4000 g at 4 °C for 30 min. Next, 135 μL of supernatant was transferred to a new 96-well plate with 10 μL of internal standard per well. Serial dilutions of derivatized stock standards were added to the remaining wells. Finally, the plates were sealed for LC-MS analysis.

Metabolite profiling and data processing were performed using an ultra-performance liquid chromatography coupled to tandem mass spectrometry (UPLC-MS/MS) system (ACQUITY UPLC-Xevo TQ-S, Waters Corp., Milford, MA, USA). The analytes were separated on an ACQUITY UPLC BEH C18 1.7-μM VanGuard pre-column (2.1×5 mm) and analytical column (2.1×100 mm). Mobile phases were used as carried liquid at a constant flow rate of 0.4 mL/min. The source and desolvation temperatures were set at 150 °C and 500 °C, respectively. Each sample was analyzed via UPLC-MS/MS in both negative and positive ionization modes to acquire the metabolite profiles.

The raw data files generated by UPLC-MS/MS were processed using MassLynx software (version 4.1, Waters Corp.) to perform peak integration, calibration, and quantitation for each metabolite. The analysis order of all test samples was randomized. Quality control (QC) samples were obtained by mixing a small aliquot of each biological sample in the study set. The pooled QC samples represented both the sample matrix and metabolite compositions of the samples. The raw pooled QC mixtures were used to produce multiple QC samples that were analyzed during the whole injection sequence. In metabolomics, application of QC samples provides a mechanism to evaluate the quality and assess the analytical variance of the acquired data. The self-developed platform, iMAP (version 1.0, Metabo-Profile, Shanghai, China), was used for statistical analyses, including principal component analysis (PCA), partial least square-discriminant analysis (PLS-DA), univariate analysis, and pathway analysis.The metabolite-pathway enrichment analysis was performed by company (Metabo-Profile, Shanghai, China) using Pathway-associated metabolite sets (Pathway-associated metabolite sets, SMPDB), while the metabolites enriched in each group were analyzed by LDA Effect Size (LEfSe) analysis^7^. Log 10 (LDA score) >2 was defined as “significance.

## **12. Metagenomic sequencing and analysis**

The DNA was extracted from cecal contents of mice were using DNeasy PowerSoil Pro Kit (Qiagen) following manufacturer protocol. Metagenomic libraries were constructed from 100–250 pg of DNA using the Nextera XT DNA Library Preparation Kit (Illumina) as per the manufacturer’s recommended protocol and sequenced on the HiSeq 2500 2x101 PE platform (Illumina) by commercial company (Personal Biotechnology Co.,Ltd., Shanghai). The raw metadata was filtered using fastp v 0.20.0^8^, and was annotated by kraken2 with a customized database as described in our previous study^1^ and the relative abundance (RA) of each species was extracted by bracken. The taxonomy annotation and RA was tabulated and the taxonomy with RA> 10-5 was used for further analysis. The α-diversity and β-diversity was analyzed by MicrobiomeAnalyst with default parameters^9^. The species enriched in each group was analyzed by LEfSe analysis^7^. The function of metagenomes was annotated using KEGG database at different KEGG orthology (KO) hierarchies^10^. The abundances of KEGG pathways were tabulated, and the enrichment of ko pathway in each group was determined with LEfSe analysis. For the analysis of metagenomes and metabolome, the cladogram was generated with Graphlan v1.1.3^11^. The Sankey diagrams were generated with RAWGraphs 2.0, while the PCoAs, Bar charts and Heatmaps were generated with ImageGP^12^. All the diagrams for each figure were composed and typeset with Adobe Illustrator CC 2018. The correlation network between group-enriched metabolites and gut microbes were analyzed using 3MCor website^13^, and the pairwise spearman correlations (r>0.6) between metabolites and gut microbial species were used for further visualization with Cytoscape v3.9.1.

## **13. Statistical analysis**

The normality of data distribution was assessed with a Kolmogorov-Smirnov test. For comparisons between > 2 groups, one-way ANOVA followed by Turkey's posthoc test, and two-way ANOVA followed by the Bonferroni post hoc correction; for comparisons between 2 groups, unpaired two-tailed Student's t-test (parametric, two groups). To analyze specific microbiota changes, univariate analyses of selected species (after centered log-ratio transformation) were performed by Kruskal-Wallis ANOVA with Dunn’s post-hoc test. To analyze the association between two factors, spearman correlation analysis were performed. Above tests were conducted with GraphPad Prism version 9.0 (GraphPad Software, San Diego, CA). Significant differences were indicated by **P* < 0.05, ***P* < 0.01, ****P* < 0.001,*****P* < 0.0001. *P* -value between 0.05 and 0.1 indicated a trend toward a significant effect. To acquire unbiased data, the investigator who administered the treatment was the only person aware of the treatment group allocation. Another technician and research assistant evaluated the biochemical analysis, who were blind to the types of treatments.

# **Supplementary figures**

**
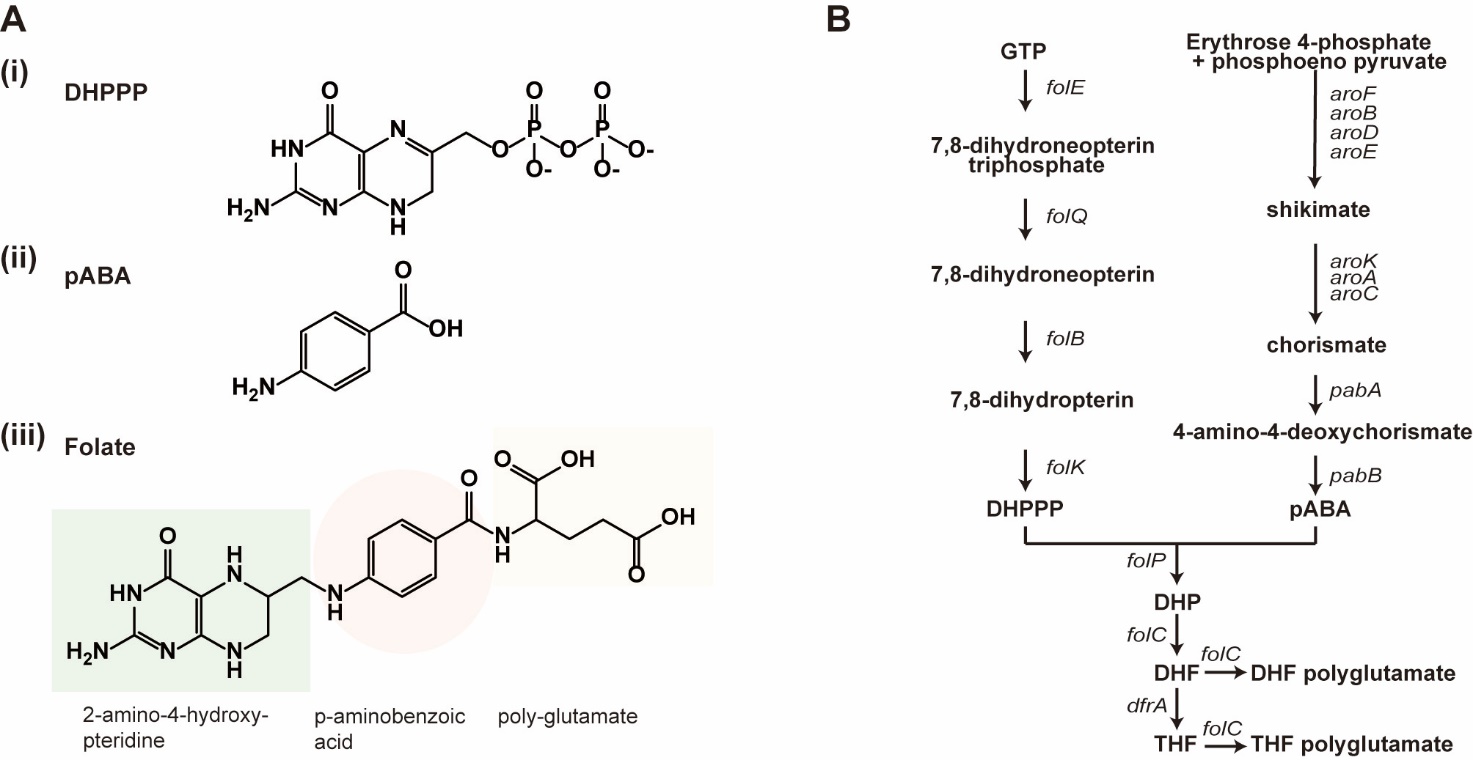
**

**Figure S1. The chemical structure of the folate and schematic of bacterial *de novo* folate synthesis. corresponding to Fig. 1.**

**A**. Chemical structure of the folate family, chemically, folates consist of three distinct chemical moieties linked together. (**i**) A pterin (2-amino-4-hydroxy-pteridine) heterocyclic ring is linked by a methylene bridge to a (**ii**) p-aminobenzoyl group that in turn is bonded through an amide linkage to (**iii**) either glutamic acid or poly-glutamate.

**B**. The schematic of bacterial *de novo* folate synthesis.


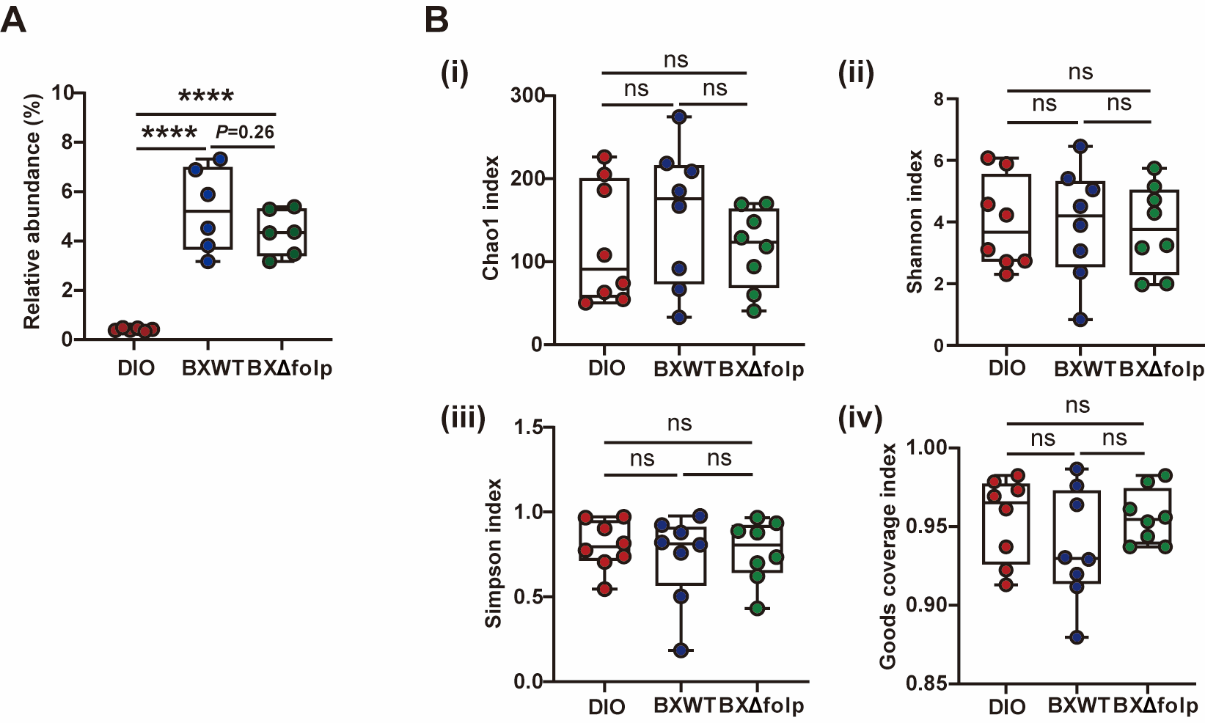


## **Figure S2. The relative abundance and** **α-diversity of gut microbiota in *B. xylanisolvens* wild-type or *folp* mutant colonized DIO mice,** **corresponding to Fig. 1.**

**A.** The relative abundance of *B. xylanisolvens* in different groups.

**B.** The α-diversity indexes ((**i**) chao 1 index; (**ii**) shannon index; (**iii**) simpson index; (**iii**) good coverage index) of gut microbiota in different groups.

Abbreviation: DIO, high fat diet-induced obese mice; BXWT, *B. xylanisolvens*; BXΔfolP, *B. xylanisolvens* with knockout of *folP* gene*.* In **A** and **B**, data are shown as mean ± SEM. Each dot represents one mouse. Statistical analysis was performed using one-way ANOVA with Tukey's test; *****P* <0.0001, ****P* <0.001,***P* <0.01, **P* <0.05, ns, not significant.

**
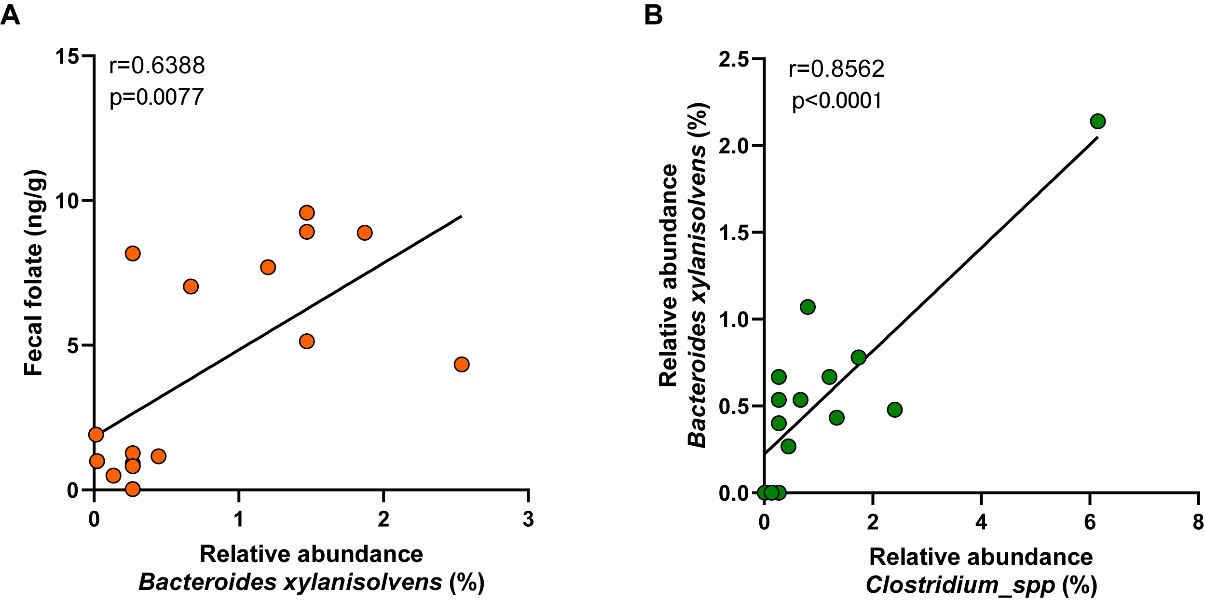
**

**Figure S3. The spearman correlation analysis of fecal folate-*B. xylanisolvens*, and *B. xylanisolvens*-*Clostridium spp.*, corresponding to Fig. 1.**

**A.** The spearman correlation analysis of fecal folate-*B. xylanisolvens*, data from DIO (high fat diet-induced obese mice) and BXWT (*B. xylanisolvens*) groups;

**B.** The spearman correlation analysis of *B. xylanisolvens*-*Clostridium spp.*( sum relative abundance of four species, *Clostridium_sp_Culture-41*; *Clostridiales_bacterium_CIEAF_016*; *unidentified_Clostridiales*; *Clostridium_sp_Culture-54*), data from DIO (high fat diet-induced obese mice) and BXWT (*B. xylanisolvens*) groups.

Each dot represents one mouse. Statistical analysis was performed using spearman correlation analysis.


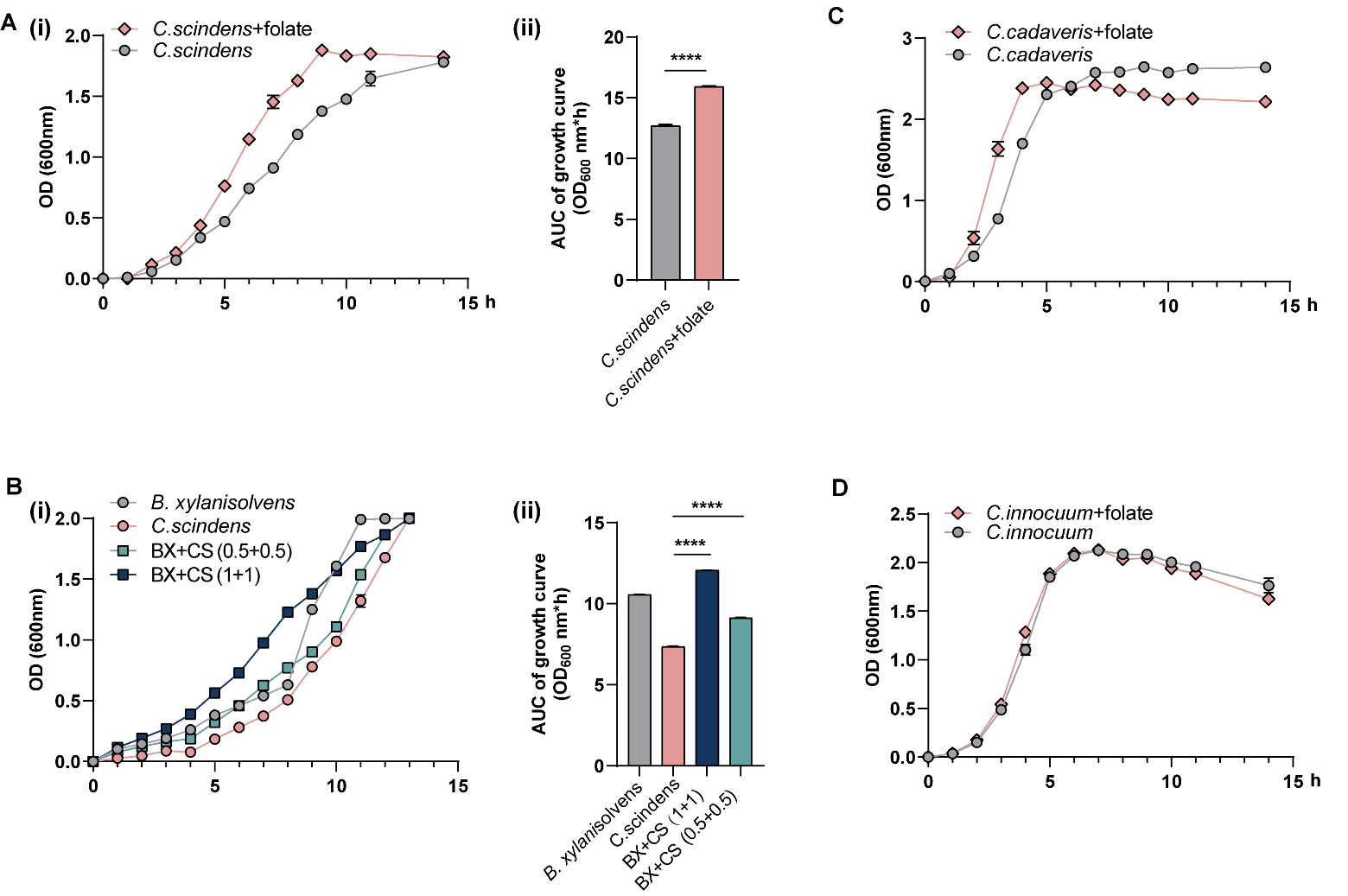


**Figure S4. Exploration of cross-feeding between *Bacteroides xylanisolvens* and *Clostridium spp.*, corresponding to Fig. 2.**

**A.** The growth curve (**i**) and area under the curve (AUC) (**ii**) under two groups (*C. scindens* was growth in YCFA medium, the initial cell density 1×10^6^ cfu/mL; *C. scindens*  was growth in 10 μg/L folate YCFA medium, the initial cell density 1×10^6^ cfu/mL, respectively), n = 3..

**B.** The growth curve (**i**) and area under the curve (AUC) (**ii**) under four groups (*B. xylanisolvens* was growth in YCFA medium, the initial cell density 1×10^6^ cfu/mL; *C. scindens* was growth in YCFA medium, the initial cell density 1×10^6^ cfu/mL; BX+CS (0.5+0.5), *B. xylanisolvens* and *C. scindens* was growth in YCFA medium, the initial cell density 5×10^5^ cfu/mL and 5×10^5^ cfu/mL, respectively; BX+CS (1+1), *B. xylanisolvens* and *C. scindens* was growth in YCFA medium, the initial cell density 1×10^6^ cfu/mL and 1×10^6^ cfu/mL, respectively), n = 3.

**C.** The growth curve under two groups (*C. cadaveris* was growth in YCFA medium, the initial cell density 1×10^6^ cfu/mL; *C. cadaveris*  was growth in 10 μg/L folate YCFA medium, the initial cell density 1×10^6^ cfu/mL, respectively), n = 3.

**D.** The growth curve under two groups (*C. innocuum* was growth in YCFA medium, the initial cell density 1×10^6^ cfu/mL; *C. innocuum*  was growth in 10 μg/L folate YCFA medium, the initial cell density 1×10^6^ cfu/mL, respectively), n = 3.

Data in (**A**)-(**D**) are shown as mean ± SEM. Statistical analysis was performed using one-way ANOVA with Tukey's test in **Bii**, unpaired two-tailed Student's t-test in **Aii**, *****P* <0.0001, ****P* <0.001,***P* <0.01, **P* <0.05.

**
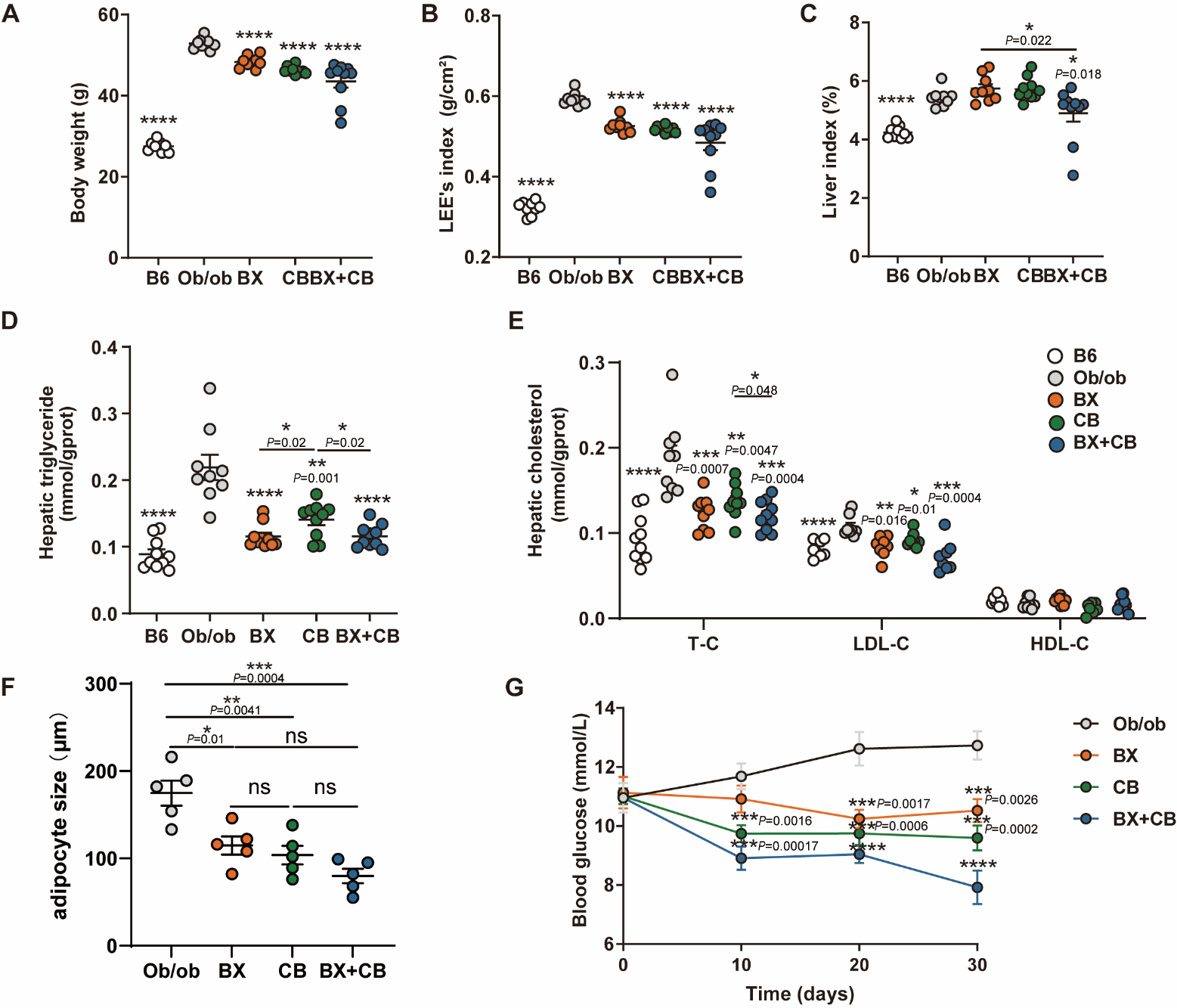
**

## **Figure S5. Synergistical reinforcement of *in vivo* anti-metabolic syndrome effect of *B. xylanisolvens* and *C. butyricum* combination therapy, corresponding to Fig. 3.**

**A.** Mouse body weight on day 35, related to **Fig. 3E**.

**B.** LEE’s index (mouse liver weight/body length^2^).

**C.** Liver index (mouse body weight/body weight × 100%).

**D.** Hepatic triglyceride.

**E.** Hepatic cholesterol.

**F.** Adipocyte size, related to **Fig. 3G**.

**G.** Blood glucose at different time points and mice were *ad libitum* feeding.

Abbreviation: B6, negative control group, *C57BL/6J* mice as the wild-type genotype background of *ob/ob* mice; *ob/ob*, model group, PBS-treated *ob/ob* mice; BX, *B. xylanisolvens*-treated *ob/ob* mice; CB, *C. butyricum*-treated *ob/ob* mice; BX+CB, *B. xylanisolvens* and *C. butyricum*-treated *ob/ob* mice.

In **A**-**F**, data are shown as mean ± SEM. Each dot represents one mouse. Statistical analysis was performed using one-way ANOVA with Tukey's test; two-way ANOVA followed by the Bonferroni post hoc correction in **G**, *****P* <0.0001, ****P* <0.001, ***P* <0.001, **P* <0.05.


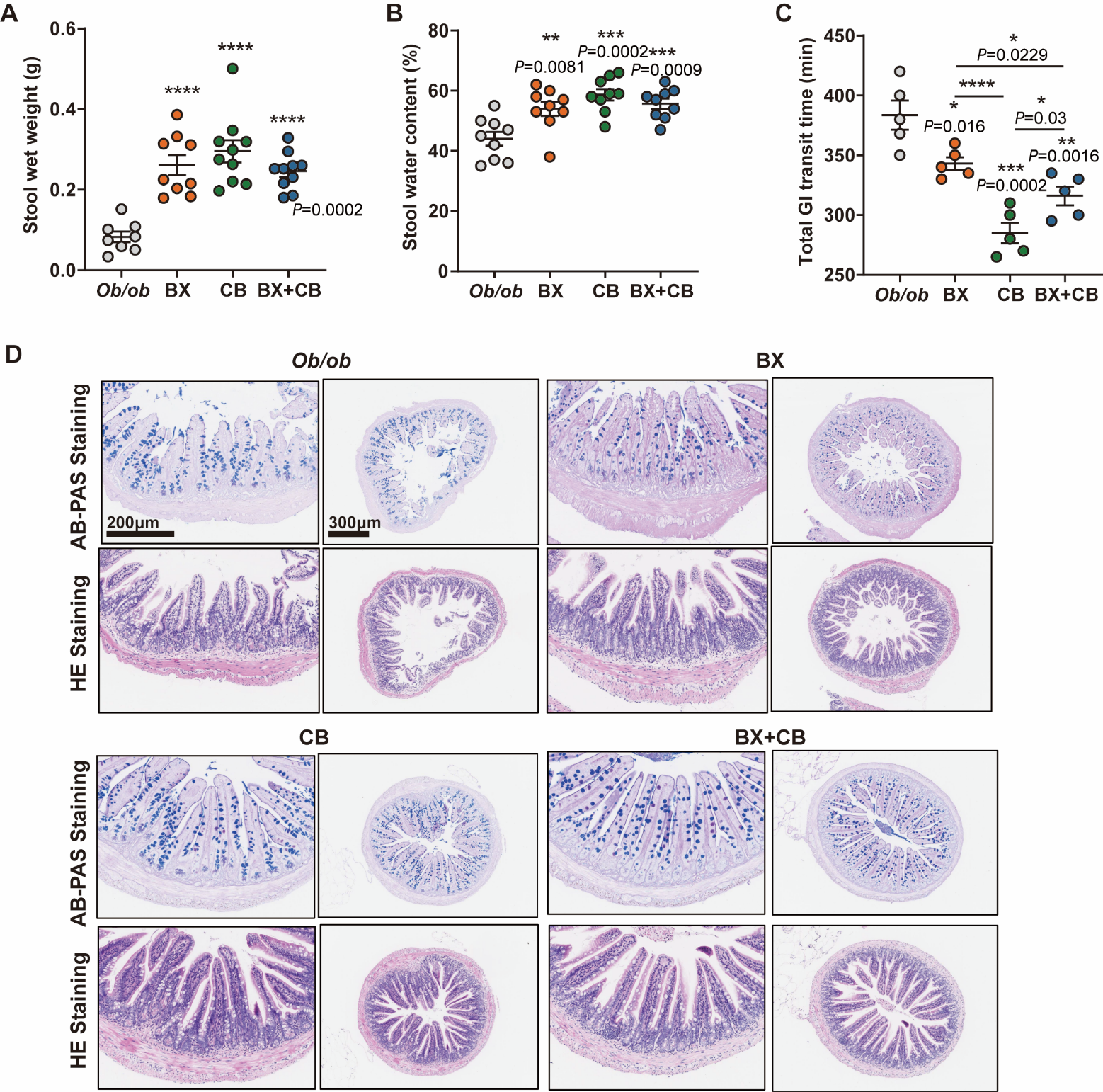


## **Figure S6.** **Synergistical reinforcement of *in vivo* fecal characters and gastrointestinal motility function of *B. xylanisolvens* and *C. butyricum* combination therapy, corresponding to Fig. 3.**

**A**. Stool wet weight.

**B**. Stool water content.

**C**. Total GI transit time.

**D**. The representative ileum pathological sections by HE or AB-PAS staining among different groups; scale bar, 200 μm or 300 μm.

Abbreviation: *ob/ob*, model group, PBS-treated *ob/ob* mice; BX, *B. xylanisolvens*-treated *ob/ob* mice; CB, *C. butyricum*-treated *ob/ob* mice; BX+CB, *B. xylanisolvens* and *C. butyricum*-treated *ob/ob* mice.

In **A**-**C**, data are shown as mean ± SEM. Each dot represents one mouse. Statistical analysis was performed using one-way ANOVA with Tukey's test, *****P* <0.0001, ****P* <0.001, ***P* <0.001, **P* <0.05.

**
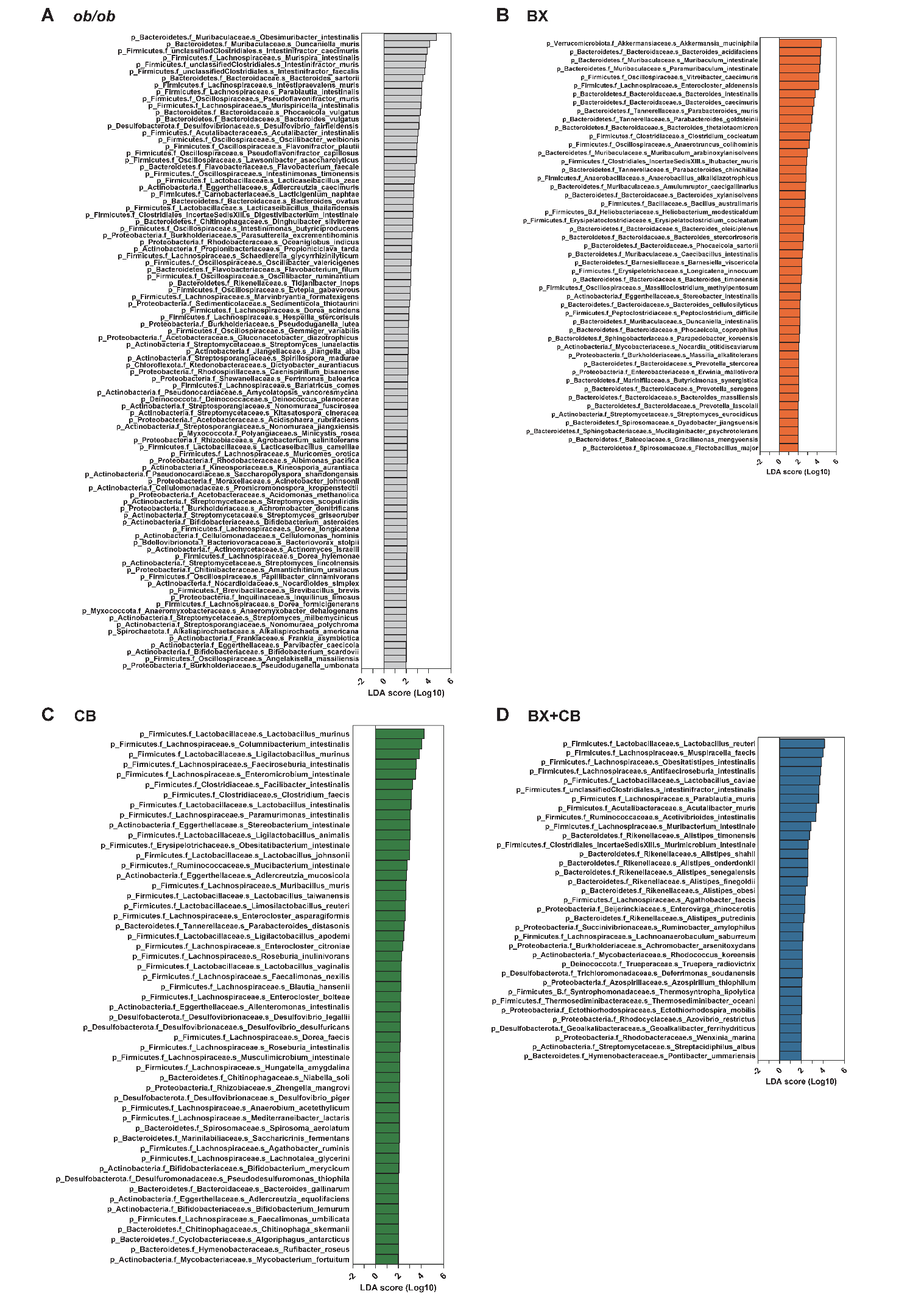
**

## **Figure S7.** **The enriched species in each group by LEfSe analysis, corresponding to Fig. 4.**

**A**. The linear discriminant analysis (LDA) score of enriched species in *ob/ob* group.

**B**. The LDA score of enriched species in BX group.

**C**. The LDA score of enriched species in CB group.

**D**. The LDA score of enriched species in BX+CB group.

Abbreviation: *ob/ob*, model group, PBS-treated *ob/ob* mice; BX, *B. xylanisolvens*-treated *ob/ob* mice; CB, *C. butyricum*-treated *ob/ob* mice; BX+CB, *B. xylanisolvens* and *C. butyricum*-treated *ob/ob* mice.

**
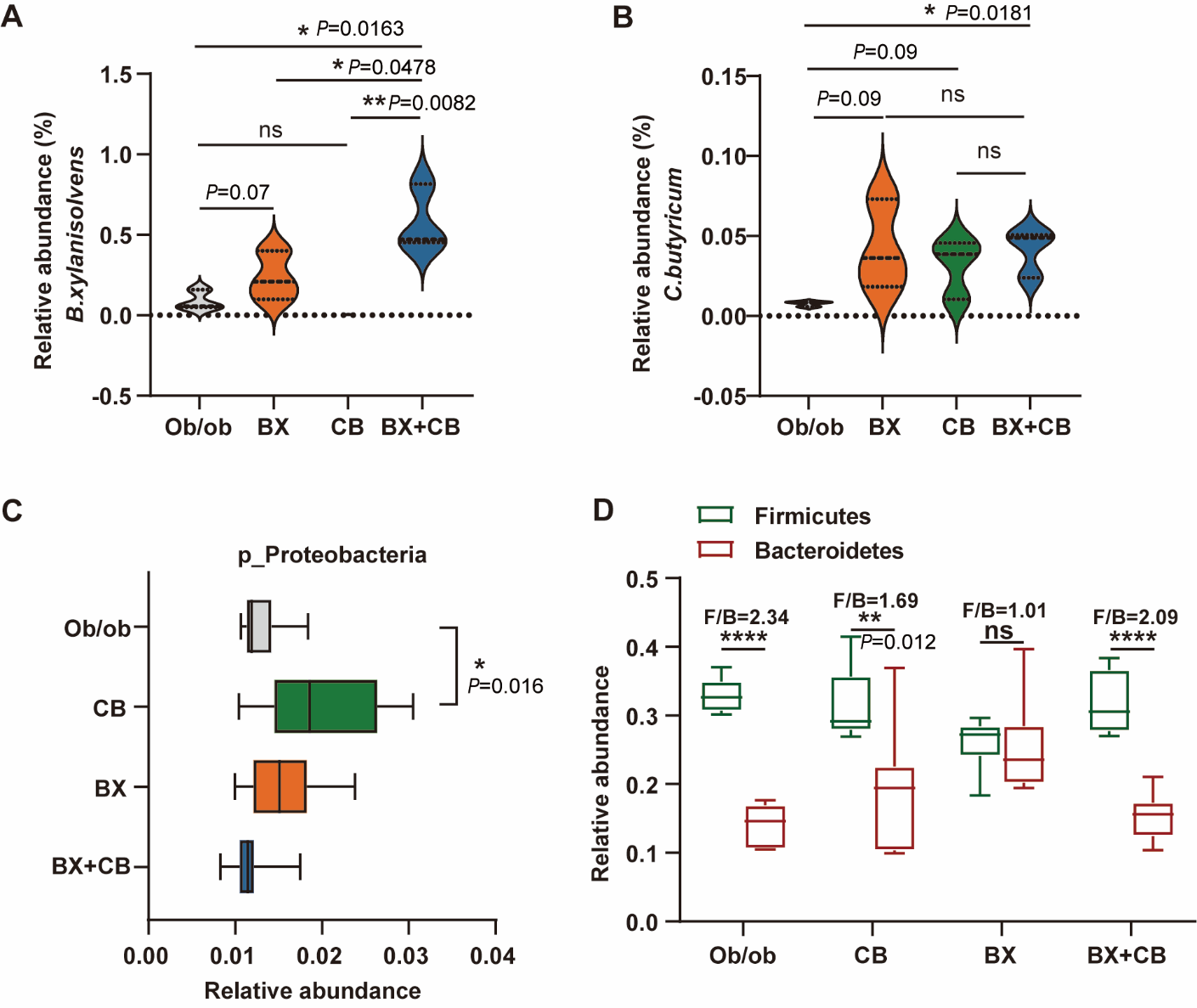
**

## **Figure S8. The relative abundance of *B. xylanisolvens***, ***C. butyricum***, **and Proteobacteria in each group.**

**A**. The relative abundance of *B. xylanisolvens*.

**B**. The relative abundance of *C. butyricum*.

**C**. The relative abundance of Proteobacteria.

**D**. The relative abundance of Firmicutes and Bacteroidetes.

Abbreviation: *ob/ob*, model group, PBS-treated *ob/ob* mice; BX, *B. xylanisolvens*-treated *ob/ob* mice; CB, *C. butyricum*-treated *ob/ob* mice; BX+CB, *B. xylanisolvens* and *C. butyricum*-treated *ob/ob* mice. Statistical analysis was performed using unpaired two-tailed Student's t-test, *****P* <0.0001, ****P* <0.001, ***P* <0.001, **P* <0.05, ns, not significant.

**
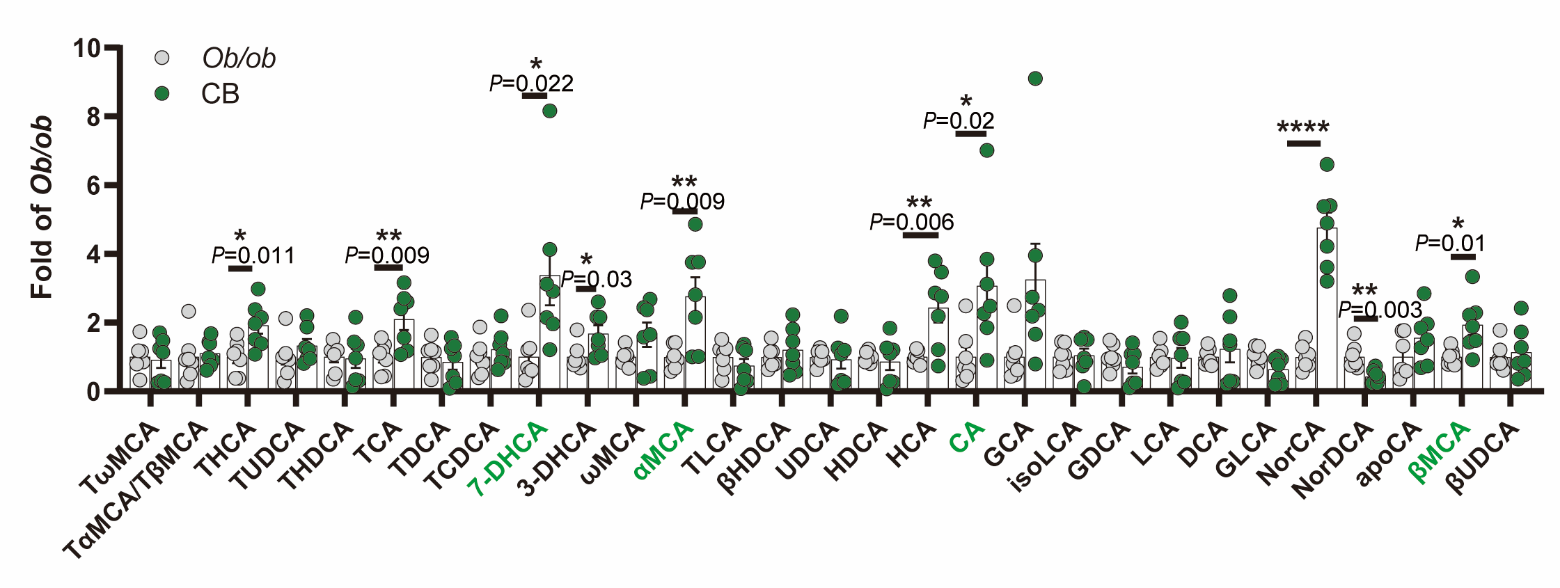
**

## **Figure S9. The fecal bile acids profiling of *ob/ob* mice treatment with PBS or** ***C. butyricum*, corresponding to Fig. 7.**

Each dot represents one mouse. Statistical analysis was performed using unpaired two-tailed Student's *t*-test, *****P* <0.0001, ****P* <0.001, ***P* <0.001, **P* <0.05.

Abbreviation: *ob/ob*, model group, PBS-treated *ob/ob* mice; CB, *C. butyricum*-treated *ob/ob* mice.

**References:**

1. Liu C, Du M-X, Abuduaini R, et al. Enlightening the taxonomy darkness of human gut microbiomes with a cultured biobank. Microbiome. 2021;9(1):119. doi:10.1186/s40168-021-01064-3

2. Olson CA, Vuong HE, Yano JM, Liang QY, Nusbaum DJ, Elaine Y. Hsiao. The Gut microbiota mediates the anti-seizure effects of the ketogenic diet. Cell. 2018;173(7):1728-1741.e13. doi:10.1016/j.cell.2018.04.027

3. Wang K, Liao M, Zhou N, et al. Parabacteroides distasonis alleviates obesity and metabolic dysfunctions via production of succinate and secondary bile acids. Cell Reports. 2019;26(1):222-235.e5. doi:10.1016/j.celrep.2018.12.028

4. Qiao S, Bao L, Wang K, et al. Activation of a specific gut Bacteroides-folate-liver axis benefits for the alleviation of nonalcoholic hepatic steatosis. Cell Reports. 2020;32(6):108005. doi:10.1016/j.celrep.2020.108005

5. Masella AP, Bartram AK, Truszkowski JM, Brown DG, Neufeld JD. PANDAseq: paired-end assembler for illumina sequences. BMC Bioinformatics. 2012;13(1):31. doi:10.1186/1471-2105-13-31

6. Love MI, Huber W, Anders S. Moderated estimation of fold change and dispersion for RNA-seq data with DESeq2. Genome Biol. 2014;15(12):550. doi:10.1186/s13059-014-0550-8

7. Segata N, Izard J, Waldron L, et al. Metagenomic biomarker discovery and explanation. Genome Biol. 2011;12(6):R60. doi:10.1186/gb-2011-12-6-r60

8. Chen S, Zhou Y, Chen Y, Gu J. fastp: an ultra-fast all-in-one FASTQ preprocessor. Bioinformatics. 2018;34(17):i884-i890. doi:10.1093/bioinformatics/bty560

9. Chong J, Liu P, Zhou G, Xia J. Using microbiome analyst for comprehensive statistical, functional, and meta-analysis of microbiome data. Nat Protoc. 2020;15(3):799-821. doi:10.1038/s41596-019-0264-1

10. Mao X, Cai T, Olyarchuk JG, Wei L. Automated genome annotation and pathway identification using the KEGG Orthology (KO) as a controlled vocabulary. Bioinformatics. 2005;21(19):3787-3793. doi:10.1093/bioinformatics/bti430

11. Asnicar F, Weingart G, Tickle TL, Huttenhower C, Segata N. Compact graphical representation of phylogenetic data and metadata with GraPhlAn. PeerJ. 2015;3:e1029. doi:10.7717/peerj.1029

12. Chen T, Liu Y, Huang L. ImageGP: An easy‐to‐use data visualization web server for scientific researchers. iMeta. 2022;1(1):e5. doi:10.1002/imt2.5

13. Sun T, Li M, Yu X, et al. 3MCor: an integrative web server for metabolome–microbiome-metadata correlation analysis Martelli PL, ed. Bioinformatics. 2022;38(5):1378-1384. doi:10.1093/bioinformatics/btab818
